# A novel *Botrytis cinerea‐*specific gene *BcHBF1* enhances virulence of the grey mould fungus via promoting host penetration and invasive hyphal development

**DOI:** 10.1111/mpp.12788

**Published:** 2019-04-22

**Authors:** Yue Liu, Jiane‐Kang Liu, Gui‐Hua Li, Ming‐Zhe Zhang, Ying‐Ying Zhang, Yuan‐Yuan Wang, Jie Hou, Song Yang, Jiao Sun, Qing‐Ming Qin

**Affiliations:** ^1^ College of Plant Sciences Key Laboratory of Zoonosis Research, Ministry of Education, Jilin University Changchun 130062 China; ^2^ College of Plant Sciences Jilin University Changchun 130062 China; ^3^ Department of Forest Forest College of Beihua University Jilin 132013 China; ^4^ Present address: College of Life Sciences Tsinghua University Beijing 100084 China

**Keywords:** appressorium, *Botrytis cinerea*, host penetration, hyphal branching, infection cushions, sclerotial formation, virulence

## Abstract

*Botrytis cinerea* is the causative agent of grey mould on over 1000 plant species and annually causes enormous economic losses worldwide. However, the fungal factors that mediate pathogenesis of the pathogen remain largely unknown. Here, we demonstrate that a novel *B. cinerea*‐specific pathogenicity‐associated factor *BcHBF1 *(hyphal branching‐related factor 1), identified from virulence‐attenuated mutant M8008 from a *B. cinerea* T‐DNA insertion mutant library, plays an important role in hyphal branching, infection structure formation, sclerotial formation and full virulence of the pathogen. Deletion of *BcHBF1* in *B. cinerea* did not impair radial growth of mycelia, conidiation, conidial germination, osmotic‐ and oxidative‐stress adaptation, as well as cell wall integrity of the ∆*Bchbf1* mutant strains. However, loss of *BcHBF1* impaired the capability of hyphal branching, appressorium and infection cushion formation, appressorium host penetration and virulence of the pathogen. Moreover, disruption of *BcHBF1 *altered conidial morphology and dramatically impaired sclerotial formation of the mutant strains. Complementation of *BcHBF1 *completely rescued all the phenotypic defects of the ∆*Bchbf1* mutants. During young hyphal branching, host penetration and early invasive growth of the pathogen, *BcHBF1* expression was up‐regulated, suggesting that *BcHBF1 *is required for these processes. Our findings provide novel insights into the fungal factor mediating pathogenesis of the grey mould fungus via regulation of its infection structure formation, host penetration and invasive hyphal branching and growth.

## Introduction

*Botrytis cinerea* is a typical necrotrophic plant fungal pathogen that inflicts grey mould on over 1000 plant species (Fillinger and Elad, 2016), including almost all vegetable and fruit crops, and annually causes US$10 billion to US$100 billion in losses worldwide (Weiberg *et al*., 2013). In the field, the main infection source of the pathogen is conidia that germinate on plant surfaces and form appressorium‐like structures to facilitate host penetration (Gourgues *et al*., 2004). Mycelia of the pathogen are able to form highly melanized ‘specialized hyphal networks’ or ‘clumps of hyphae’ called infection cushions that also promote the pathogen host invasion (Cao *et al*., 2018; Liu *et al*., 2018; Marschall and Tudzynski, 2016). Besides infection structures (appressoria and infection cushions), occasionally, host invasion by the pathogen can occur via germ tube apices (Choquer *et al*., 2007; Van den Heuvel and Waterreus, 1983). *B. cinerea* induces a very wide range of symptoms; the most typical symptoms on leaves and soft fruits are soft rots, accompanied by collapse and water soaking of parenchyma tissues, followed by a rapid appearance of grey masses of conidia that initiate the next round of infection. *B. cinerea* produces sclerotia that develop within dying host tissues and serve as the long‐term survival structures in the life cycle and the primary inoculum in the disease cycle. The pathogen can also survive as mycelia, surviving within crop debris and inside some seeds to serve as primary inoculum. Sclerotia germinate in early spring to produce conidiophores and multinucleate conidia, serving as a primary source of inoculum (Williamson *et al*., 2007).

Host infection by *B. cinerea* is complicated and tightly regulated by numerous pathogenicity‐associated factors including extracellular enzymes, proteins and metabolites. To secure a successful host infection, *B. cinerea *conidia need to germinate and form appressoria to penetrate hosts. However, *B. cinerea* conidia hardly ever germinates in the presence of only water. The pathogen gluconeogenesis thus play a crucial role in the initiation of conidial germination since the process allows the pathogen to cope with the limitation of glucose and/or other carbon sources in the infection niches (Liu *et al*., 2018). *B. cinerea* genes involved in autophagy, a mechanism of the cell that disassembles and recycles unnecessary or dysfunctional cellular components, also greatly influence conidial germination and virulence (Liu *et al*., 2019; Ren *et al*., 2017, 2018a, b). During infection, *B. cinerea* secretes a large number of extracellular virulence components including cell wall degrading enzymes, oxidoreductases, cerato‐platanin family proteins, toxic compounds such as botrydial, oxalic acid (Frías *et al*., 2011; van Kan, 2006), and even small RNAs (Wang *et al*., 2017; Weiberg *et al*., 2013). Cell wall degrading enzymes may facilitate host penetration and the conversion of host tissue into fungal biomass, while toxic compounds and reactive oxygen species (ROS) may contribute to killing of the host cells, other proteins or molecules may participate in pathogen adhesion, signal transduction, adaptation to infection‐associated environmental stresses and modulation of host immune systems during infection. These features are regarded to contribute to the pathogen with a very broad host range; for reviews, see (Nakajima and Akutsu, 2014; Sharma and Kapoor, 2017; Williamson *et al*., 2007).

*B. cinerea *is an aggressive necrotrophic pathogen; interestingly, the fungal pathogen can also systemically colonize host plants without causing disease symptoms (van Kan *et al*., 2014; Veloso and van Kan, 2018), or sometimes remain in a long period of quiescence before rotting tissues when the host physiology changes and the environment is conducive (Williamson *et al*., 2007). Infestation by the pathogen can occur anytime from seedling to ripe product. Serious damage can also occur in any stage of seedling, development, maturity, storage and sale (Dean *et al*., 2012). Currently, grey mould control mainly relies on fungicides; however, due to the pathogen’s genetic flexibility and high evolutionary potential, there is increasing concern about the development and abundance of fungicide‐resistant *B. cinerea* field strains and the overtake of fungicide‐efficiency by the fungicide‐resistant strains (Hahn, 2014; Leroch *et al*., 2013; Romanazzi *et al*., 2016).

*B. cinerea* has been regarded as the second most important phytopathogenic fungus based on its scientific and economic significance (Dean *et al*., 2012). The availability of the pathogen’s genomic sequence and its high amenability to genetic and molecular genetic manipulation, together with its economic relevance, have contributed to *B. cinerea *being one of the most extensively studied necrotrophic fungal pathogen (Dean *et al*., 2012; Van Kan *et al*., 2017). Owing to the features of significant phenotypic variations, haploid nature, small genome size and reduced repeat content and its high amenability, *B. cinerea* has long been served as a model aggressive necrotrophic pathogen to study molecular mechanisms underlying host‐pathogen interactions (Amselem *et al*., 2011; Dean *et al*., 2012; Van Kan *et al*., 2017). The knowledge about the molecular mechanisms underlying *B. cinerea* host interactions has been greatly expanded in the last two decades due to the development of many technologies (Fillinger and Elad, 2016; Nakajima and Akutsu, 2014; Sharma and Kapoor, 2017). However, many details of *B. cinerea* pathogenesis and its disarming of host resistance still remain obscure (Fillinger and Elad, 2016).

In this study, we demonstrate that *B. cinerea* hyphal branching‐related factor 1 (BcHbf1) is a novel pathogenicity‐associated factor that is only found in *B. cinerea *genome and is required for conidial morphogenesis, hyphal branching, infection structure development, sclerotial formation and full virulence of the pathogen. Mycelium development and host invasion as well as hyphal invasive growth correspond to the up‐regulation of *BcHBF1 *expression. *BcHBF1 *is dispensable for pathogen conidiation, conidial and sclerotial germination, radial growth of mycelia and osmotic‐ and oxidative‐stress adaptation as well as cell wall integrity. Our findings provide new insights into *BcHBF1 *mediation of the development and pathogenicity of the necrotrophic fungal pathogen.

## Results

### *BcHBF1 *is a novel *B. cinerea*‐specific virulence‐associated gene

**Figure 1:**
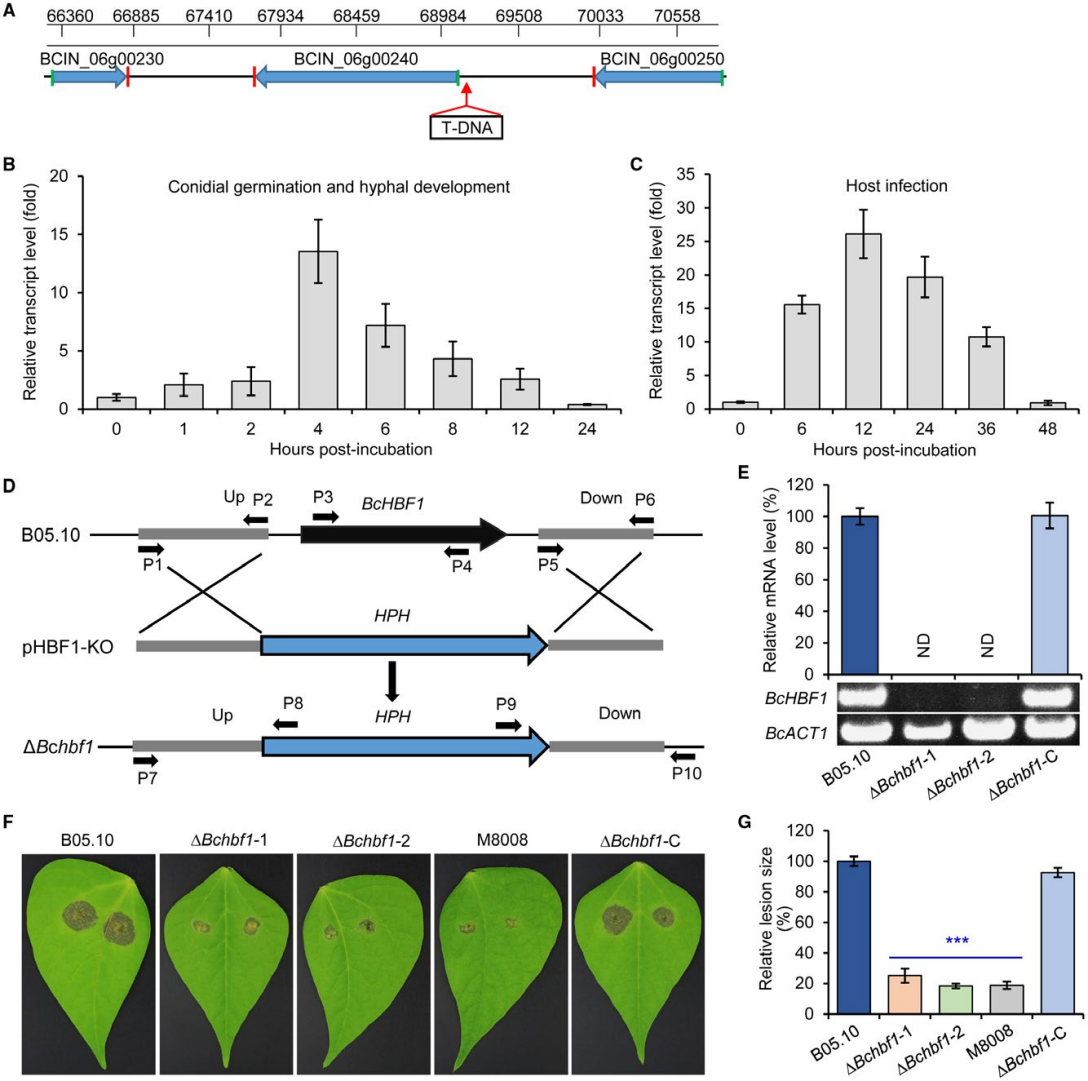
*BcHBF1* is a pathogenicity‐associated gene. (A) Schematic diagram indicates the position of T‐DNA insertion in *B. cinerea* genome of the mutant M8008. (B) Expression of *BcHBF1* in *B. cinerea* during conidial germination and the subsequent hyphal development. (C) Expression profile of *B. cinerea* *BcHBF1 *during host infection by the pathogen. (D) Strategy for generation of *BcHBF1* gene disruption (∆*Bchbf1*) mutant strains. (E) Detection of *BcHBF1* expression in the wild type (WT) (B05.10), ∆*Bchbf1 *and complemented (∆*Bchbf1*‐C) strains via real‐time quantitative Reverse Transcription‐Polymerase Chain Reaction (qRT‐PCR. (F) Pathogenicity assay for the WT, T‐DNA insertional mutant M8008, ∆*Bchbf1 *mutant and complemented strains. Droplets of conidial suspension (mixture of conidial suspension [1 × 10^6^ conidia/mL] and PDB, vol: vol = 1: 1, 5 µL) of each strain were inoculated. Diseased leaves were photographically documented at 72 h post‐inoculation/incubation (hpi) at 20 °C in dark. (G) Quantification of lesion size caused by the indicated strains at 72 hpi. Data represent means ± standard deviations (SDs) from at least three independent experiments with triplicate samples examined for each treatment. ***: significant at *P* < 0.001.

To comprehensively understand molecular mechanisms underlying *B. cinerea* host interactions, we generated a *B. cinerea* T‐DNA insertion mutant library that contains about 50 000 transformants and identified a mutant strain M8008 with a significant reduction in pathogenicity on detached tomato and strawberry leaves from the mutant library. Thermal Asymmetric Interlaced‐Polymerase Chain Reaction (TAIL‐PCR) and sequencing analysis of the mutant strain M8008 indicated that a T‐DNA inserted into the position 24 bp upstream of the start coding region of an open reading frame (ORF) (BCIN_06g00240) (Fig. 1A) that has been previously annotated as a gene encoding a hypothetical protein (Van Kan *et al*., 2017). The deduced hypothetical protein contains 465 amino acid residues (Fig. S1A) and only a few evolutionarily conserved domains, i.e. (non)cytoplasmic and transmembrane domains, were detected in the hypothetical protein (Fig. S1B). Bioinformatics analysis suggests that the hypothetical protein may be a membrane protein that contains a non‐cytoplasmic domain, a transmembrane region/TMhelix, and a cytoplasmic domain (Fig. S1B). A BLAST search demonstrated that protein orthologs of the hypothetical protein were not detected, implying that the hypothetical protein is a *B. cinerea‐*specific virulence‐associated factor. Further functional analysis suggested that the protein is required for the pathogen hyphal branching (see below), the T‐DNA tagged gene was thus designated as *BcHBF1*.

To evaluate the roles of *BcHBF1* in *B. cinerea* development and pathogenesis, we profiled *BcHBF1* expression during the pathogen conidial germination, mycelial development and host infection via real‐time quantitative Reverse Transcription (qRT‐PCR) approach. Our data demonstrated that the expression of *BcHBF1* was at a relatively low level at 1 h–2 h after the initiation of conidial germination and maintained an up‐regulated level to 4 h post‐inoculation/incubation (hpi) and reached a peak (13.54‐fold) at 4 hpi (Fig. 1B). However, after 4 hpi, the expression of *BcHBF1* gradually decreased. During a time course of 48 h of host infection, *BcHBF1* expression was up‐regulated after 6 hpi, reached a peak at 12 hpi and then back to normal level at 48 hpi (Fig. 1C). The results imply that *BcHBF1* plays an important role in the pathogen’s hyphal development and host infection.

To analyse the roles of *BcHBF1 *in the pathogen growth and virulence, we first generated *B. cinerea* *HBF1* gene knockout (KO) mutant ∆*Bchbf1* using the illustrated strategy (Fig. 1D) and its complemented strain ∆*Bchbf1‐*C as previously described (Liu *et al*., 2018). We then performed pathogenicity assays for the wild type (WT) (B05.10), T‐DNA mutant M8008, ∆*Bchbf1*, and complemented ∆*Bchbf1‐*C strains of the pathogen (conidial suspension in ½ potato dextrose broth [PDB]) after confirmation of the absence of gene *BcHBF1* in the mutant strains via PCR detection and qRT‐PCR analysis (Fig. 1E). Our result indicated that loss of *BcHBF1* significantly reduced virulence of the ∆*Bchbf1* mutants, which corresponded to pathogenicity reduction in the mutant M8008. Complementation of the mutant strain ∆*Bchbf1*‐1 with the *B. cinerea* WT *HBF1* allele rescued the pathogenicity defect of the mutants (Fig. 1F and G). These results demonstrate that *B. cinerea HBF1* is a novel virulence‐associated factor.

### *BcHBF1* is required for *B. cinerea* conidial morphogenesis but dispensable for radial growth of mycelia and conidiation

To test a role of *BcHBF1* in mediation of *B. cinerea* growth, we determined mycelial growth of the WT, Δ*Bchbf1*, and complemented strains on complete medium (CM) plates. Our results demonstrated that when mycelial plugs (Fig. S2A) or conidia (Fig. S2B) of the tested strains were inoculated on CM plates, mycelial growth (rate) of the tested strains was similar during a time course of 3 days (for mycelial plugs) or 4 days (for conidia) of incubation (Fig. S2). These results indicated that loss of *BcHBF1* in *B. cinerea* does not affect radial growth of the fungal mycelia.

**Figure 2:**
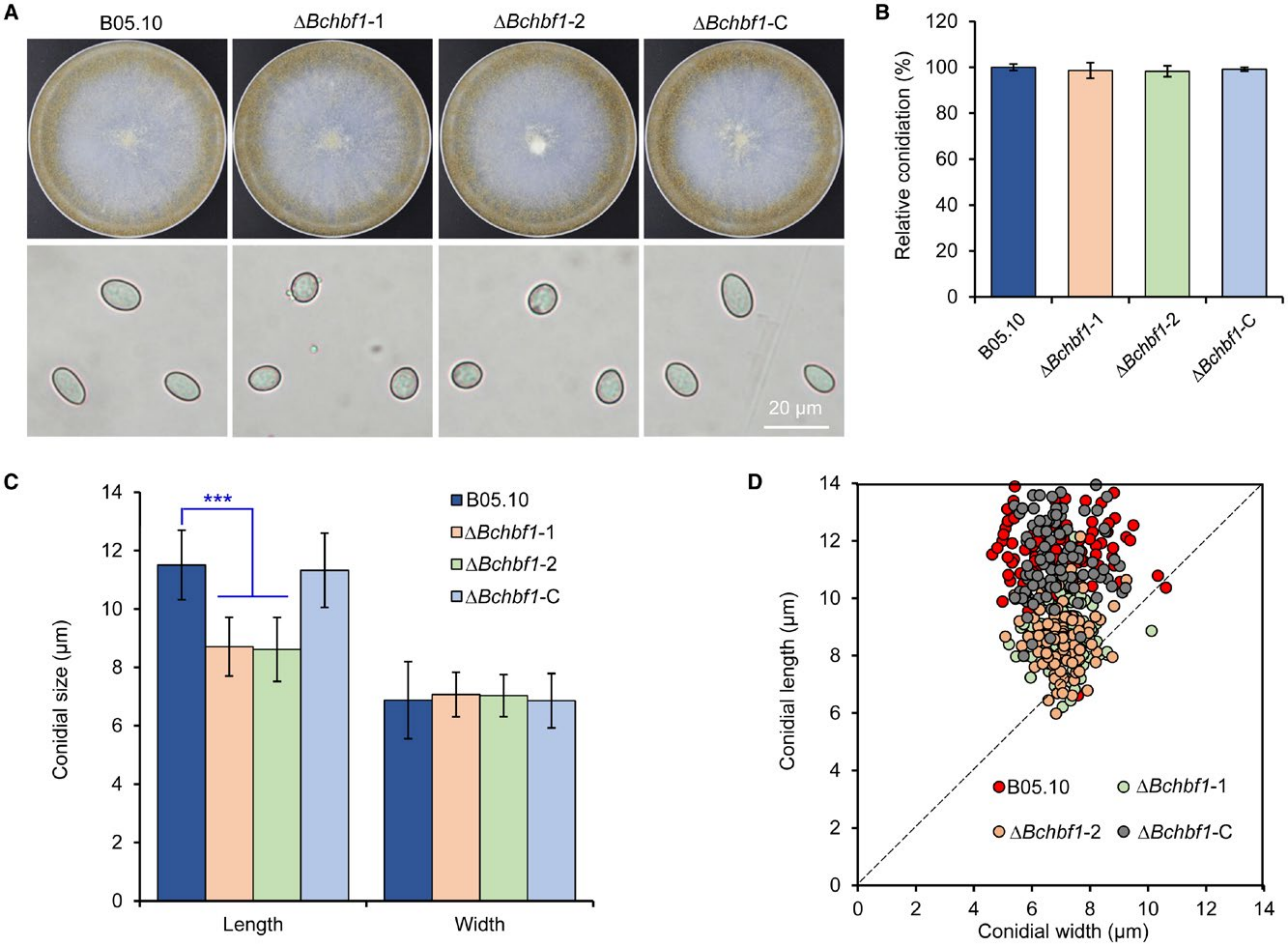
*BcHBF1* is required for *B. cinerea* conidial morphogenesis but dispensable for conidiation. (A) *BcHBF1* mediates *B. cinerea* conidiation (upper panel) and conidial morphogenesis (lower panel). The indicated *B. cinerea* WT, ∆*Bchbf1 *and complemented strains were incubated on CM plates at 20 °C for 10 days and photographically documented. (B) Quantification of relative conidiation of the indicated strains at 10 days post‐incubation/inoculation (dpi) on CM plates. (C) Comparison of conidial size of the indicated strains. (D) Loss of *BcHBF1 *increases the number of globose and less elliptical conidia (closer to the dash line) in the ∆*Bchbf1* mutant strains. More than 100 10‐day‐old conidia of each strain were measured under a microscope in each experiment. Data represent means ±  standard deviations (SDs) from three independent experiments with triplicate colonies/slides were analyzed for each strain. *** indicates significant at *P* < 0.001.

To investigate a role of *BcHBF1* in the pathogen conidiation and conidial morphogenesis, we inoculated mycelial plugs or conidia suspension of the tested strains on CM plates and determined conidiation ability and conidial morphogenesis of these strains at 10 days to 15 days post‐incubation (dpi). Our quantitative data demonstrated that loss of *BcHBF1 *did not impair conidiation of the Δ*Bchbf1 *mutant strains (Fig. 2A and B), indicating that *BcHBF1 *is dispensable for the pathogen conidiation.

Further analysis of conidial morphology revealed that the length of Δ*Bchbf1 *mutant conidia was significantly shorter than those of the WT and complemented strains (11.5 ± 1.2 μm, 8.7 ± 1.0 μm, and 11.3 ± 1.3 μm for conidia produced by the WT, Δ*Bchbf1*, and Δ*Bchbf1‐*C strains, respectively). However, conidial width of all the tested strains did not display any significant difference (6.9 ± 1.3 μm, 7.1 ± 0.8 μm, and 6.9 ± 0.9 μm for the WT, Δ*Bchbf1*, and Δ*Bchbf1‐*C strains, respectively) (Fig. 2C and D). The Δ*Bchbf1* mutant conidia are more globose and less elliptical than those of controls as demonstrated by the length‐to‐width ratio of the mutant conidia, which is closer to 1 (1.67, 1.23 and 1.65 for the WT, Δ*Bchbf1* and Δ*Bchbf1‐*C strains, respectively) (Fig. 2D). These data demonstrated that loss of *BcHBF1 *in *B. cinerea* reduced conidial size of the pathogen, although the conidial widths of the mutants were similar to those of the control and complemented strains (Fig. 2C and D). Taken together, our data demonstrate that *BcHBF1 *controls *B. cinerea* conidial morphogenesis and is dispensable for the pathogen radial growth of mycelia and conidiation.

### *BcHBF1* mediates *B. cinerea* hyphal branching but is dispensable for its conidial germination

**Figure 3:**
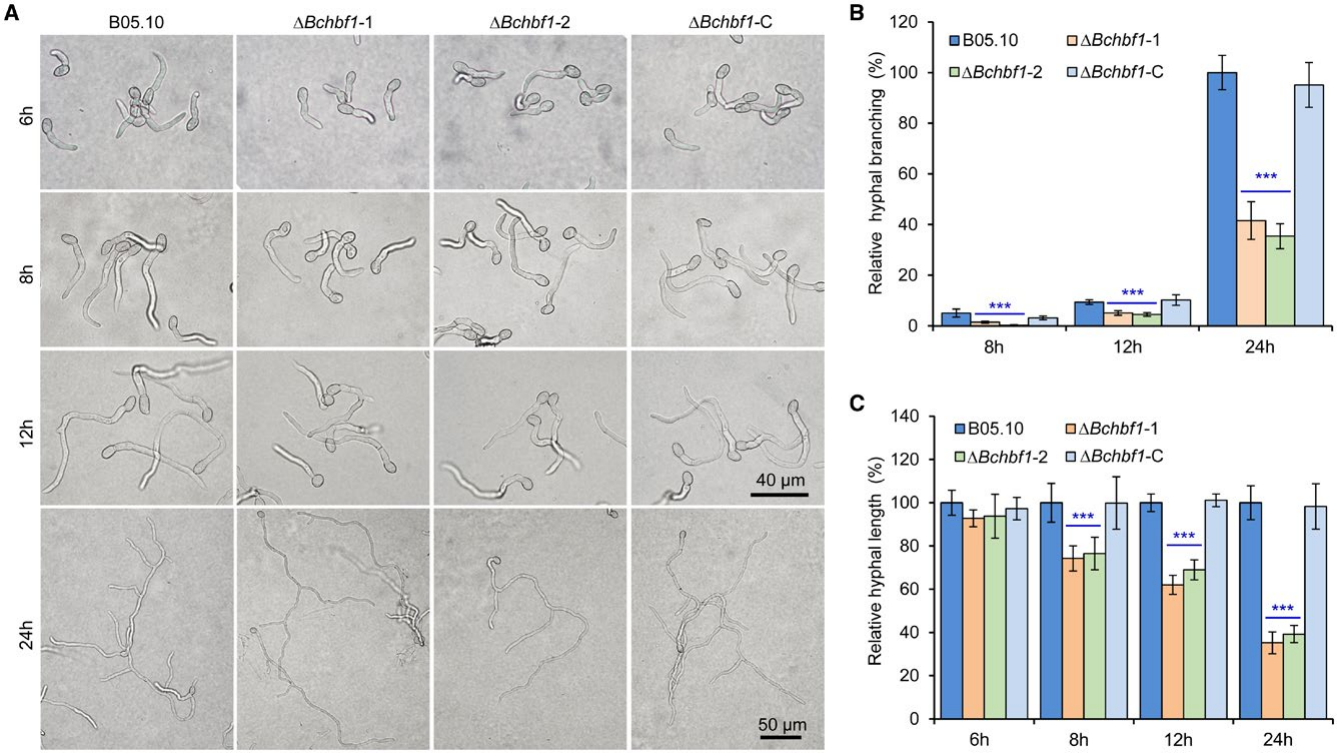
*BcHBF1* is dispensable for *B. cinerea *conidial germination but required for hyphal branching. (A) Droplets of conidial suspension (5 × 10^5^ conidia/mL, 10 µL) of each strain were inoculated on solid CM plates and incubated at 20 °C. Germinated conidia were photographically documented at the indicated hpi. (B and C) Quantification of hyphal branching (B) and total hyphal length (C) of the indicated strains at the indicated hpi. Data represent means ± standard deviations (SDs) from three independent experiments with triplicate plates examined for each treatment. ***: significant at *P* < 0.001.

To test whether *BcHBF1 *mediates *B. cinerea* conidial germination and the subsequent hyphal/mycelial development, we determine conidial germination and hyphal development on solid CM plates (Fig. 3) or on glass slides with liquid CM (Fig. S3) using the conidia harvested from CM plates at 10 dpi to 15 dpi. Our findings demonstrated that during a time course of 6 h incubation, conidial germination and germling development of the tested strains did not display significant difference (Fig. 3A and C; Fig. S3A and C). However, statistical analysis of the branched hyphae and the total hyphal length of the tested strains revealed that the difference in hyphal development between the WT and mutant strains gradually increased from 8 hpi (Fig. 3 and S3). At 14 hpi, the number of branched hyphae of the Δ*Bchbf1 *mutants was only about half of those of the WT and complemented strains (each conidium containing 2–3 and 5–6 branched hyphae in the mutant and WT strains, respectively) (Fig. S3A and B); and at 32 hpi, a similar length (~600 µm) of branched hypha contained 2–3 and 7–8 branched hyphae in the mutant and WT strains, respectively (Fig. S3A). Similar result of hyphal branching was observed when conidia of the strains cultured on solid CM at 24 hpi (Fig. 3A and B). At 8 hpi to 24 hpi, compared to the WT strain, the total hyphal length of the Δ*Bchbf1 *mutant significantly reduced (Fig. 3A and C; and Fig. S3A and C). These data demonstrate that loss of *BcHBF1* did not impair conidial germination and the germ tube development of the mutant strains, but *BcHBF1 *played an important role in mycelial development via mediating hyphal branching.

### Disruption of *BcHBF1 *impairs* B. cinerea* sclerotium formation

**Figure 4:**
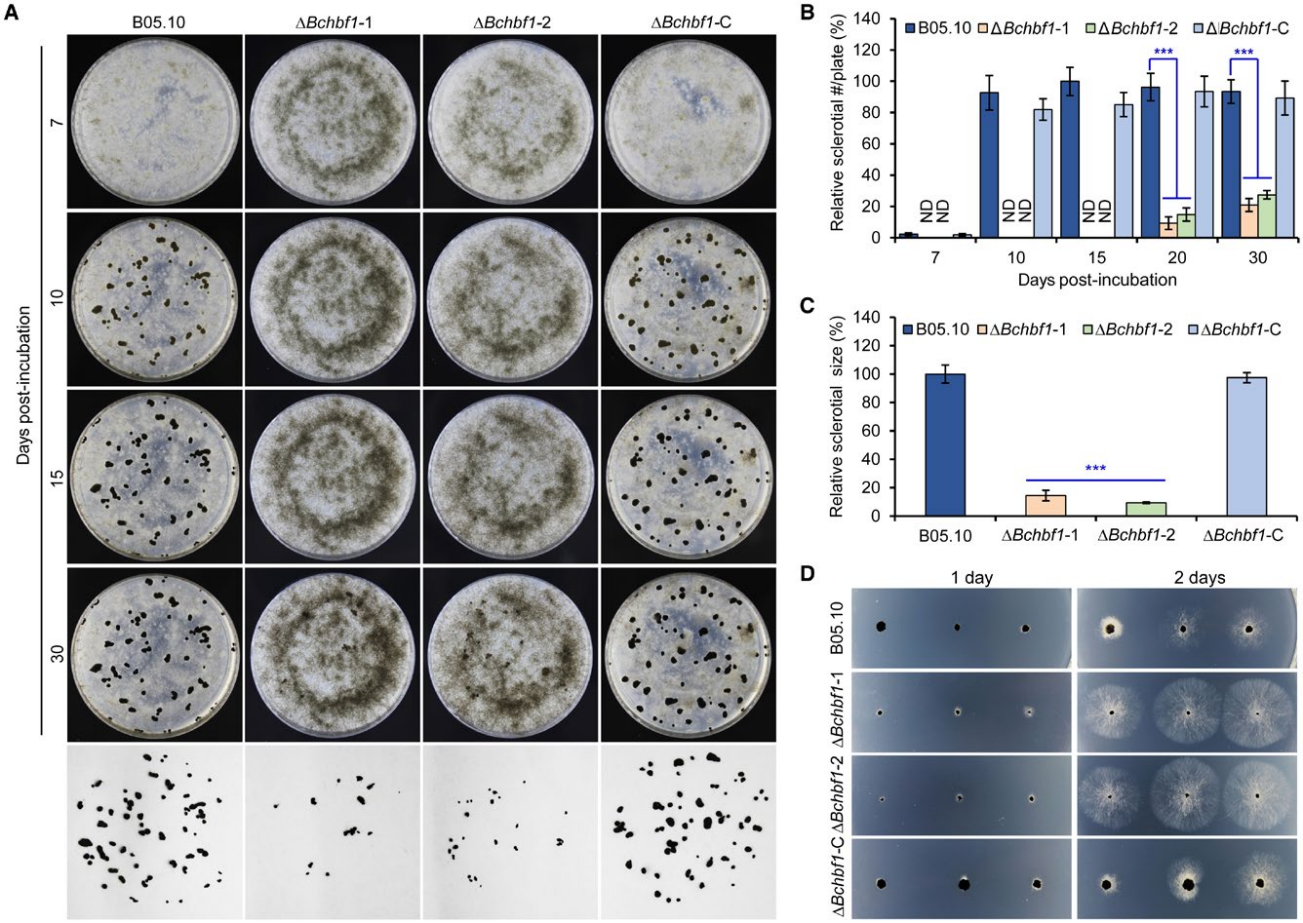
Disruption of *BcHBF1 *in *B. cinerea* impairs sclerotial production. (A) Conidia of the wild type (WT), ∆*Bchbf1*, and ∆*Bchbf1*‐C strains were inoculated on CM plates at 20 °C in darkness. Sclerotial production by each strain was observed at the indicated dpi. (B) Quantification of sclerotial formation by the indicated strains during a time course of 30 days of incubation. ND: Not detected. (C) Quantification of the sizes of sclerotia produced by the indicated strains via ImageJ (https://imagej.nih.gov/ij/). (D) Germination of sclerotia produced by the indicated strains. The representative images are from one of the experiments, at least three independent experiments were performed, and all the experiments resulted in similar results. Data represent means ±  standard deviations (SDs) from three independent experiments in which triplicate plates were analyzed for each strain in each experiment. ***: significant at *P* < 0.001.

Sclerotia play important roles in the pathogen survival in hostile environments, sexual reproduction and primary inoculum in the disease cycle (Veloso and van Kan, 2018; Williamson *et al*., 2007). To test a role of *BcHBF1* in sclerotium production, we inoculated conidia or mycelial plugs of the WT*,* Δ*Bchbf1,* and Δ*Bchbf1‐*C strains on CM plates, incubated these strains at 20 °C in dark condition, and observed and photographically documented sclerotial formation by these strains in a period of 30 days. Our results indicated that sclerotial production by the Δ*Bchbf1* strains was dramatically impaired when compared to the control and complemented strains (Fig. 4A, B and C). Disruption of *BcHBF1* delayed sclerotial formation in the mutant strains about 2 weeks (Fig. 4A and B) and sclerotia produced by the mutants reduced (*P* < 0.001) (Fig. 4A and B) and were much smaller in size (~12% of the WT control) (Fig. 4A and C). However, sclerotial germination assays indicated that these smaller sclerotia produced by the mutants did not display significant difference in germination and the subsequent mycelial growth (Fig. 4D). These findings indicate that *BcHBF1* plays an important role in *B. cinerea* sclerotial formation, but has little effect on sclerotial germination.

### *BcHBF1* is dispensable for *B. cinerea* osmotic‐ and oxidative‐stress adaptation as well as cell wall integrity

**Figure 5:**
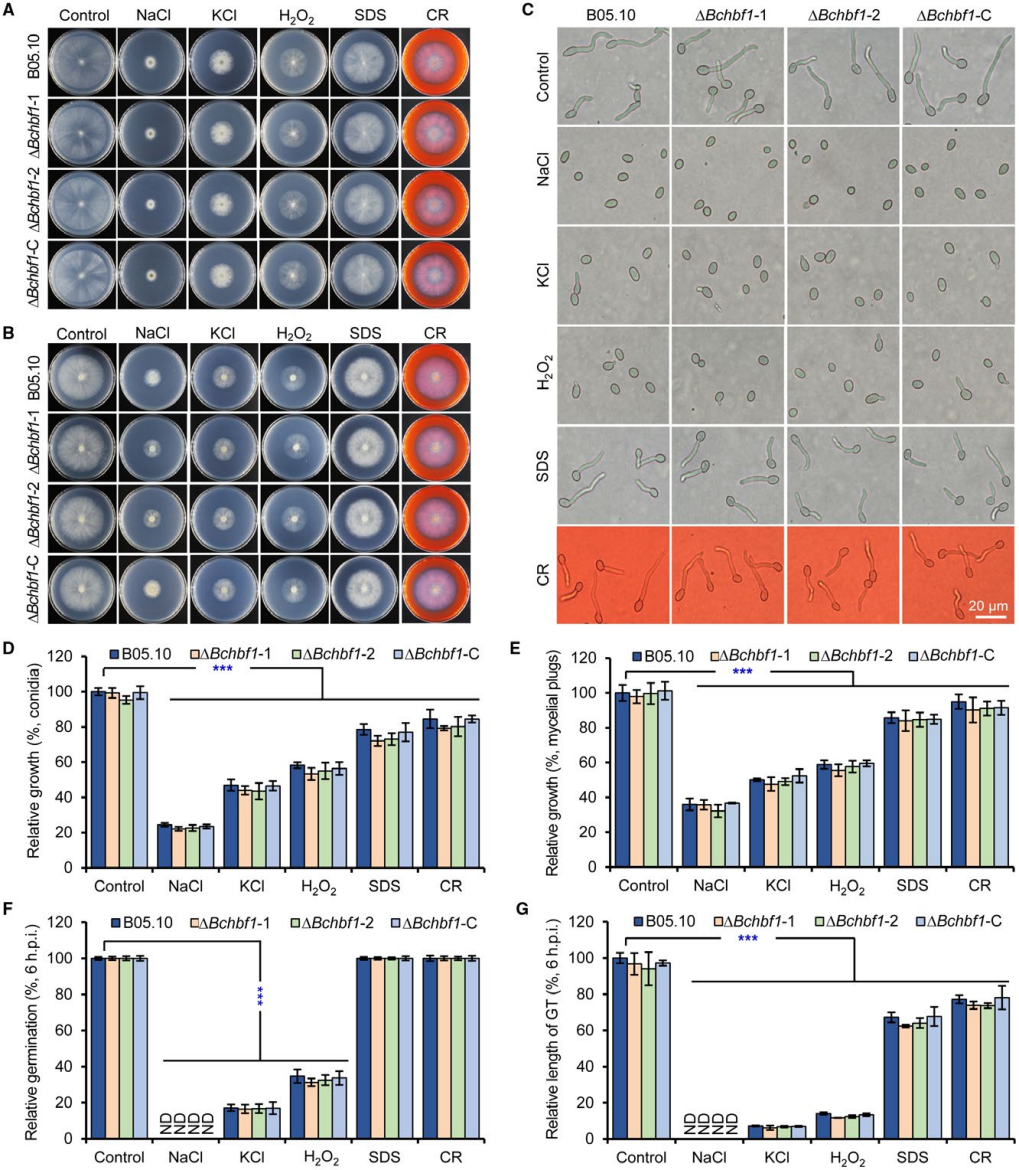
*BcHBF1* is dispensable for osmotic‐ and oxidative‐stress adaptation as well as cell wall integrity of *B. cinerea*. (A) Conidial germination (1 × 10^6^ conidia/mL, 1 µL) and hyphal development of the indicated wild type (WT), ∆*Bchbf1*, and ∆*Bchbf1*‐C strains of *B. cinerea* on CM plates supplemented with the osmotic stress agents NaCl (1 M) and KCl (1 M), the oxidative‐stress agent H_2_O_2 _(5 mM), and the cell wall disturbing agents sodium dodecyl sulfate (SDS, 0.005%) and Congo Red (CR, 300 μg/mL). (B) Mycelial radial growth (inoculated with fresh mycelial plugs, 5 mm in diameter) of the indicated strains on CM plates containing the indicated stress‐mimetic agents as presented in (A). Representative photographs were taken at 4 dpi. (C) Conidial germination (5 × 10^5^ conidia/mL, 2 µL) of the indicated strains incubated on CM plates containing the assorted stress agents as presented in (A) for 6 h. (D and E) Quantification of the relative mycelial growth of the indicated strains growing from inoculated conidia (D) or mycelial plugs (5 mm in diameter) (E) on CM plates supplemented with the indicated stress‐mimetic agents as presented in (A). (F and G) Quantification of the relative conidial germination rates (F) and germ tube development (G) of the indicated strains incubated for 6 h on CM plates containing the indicated stress agents as presented in (A). Representative images are from one experiment, at least three independent experiments were performed and all the experiments resulted in similar results. Data represent means ± standard deviations (SDs) from three independent experiments in which triplicate plates were examined for each strain in each experiment. ***: significant at *P* < 0.001.

To test whether loss of *BcHBF1* may affect *B. cinerea* adaptation to infection‐related stresses, we compared the radial growth rates of the WT, Δ*Bchbf1* and complemented strains on CM containing the osmotic stress agents NaCl and KCl, the oxidative‐stress agent H_2_O_2_, and the cell wall disturbing agents sodium dodecyl sulfate (SDS) and Congo Red (CR) (Cao *et al*., 2018; Feng *et al*., 2017; Liu *et al*., 2018). Our results demonstrated that after 4 days of incubation of conidia (Fig. 5A) or mycelial plugs (Fig. 5B) on CM containing the indicated stress‐mimetic agents, mycelial growth of all the tested strains did not display any significant difference (Fig. 5A, B, D and E), which was consistent with microscopic observation of conidial germination and germling development of these tested strains on CM containing the stress‐mimic agents (Fig. 5C, F and G). These data suggest that *BcHBF1* is dispensable for the pathogen osmotic‐ and oxidative‐stress adaptation as well as cell wall integrity*.*

### *BcHBF1* plays a crucial role in infection structure development

**Figure 6:**
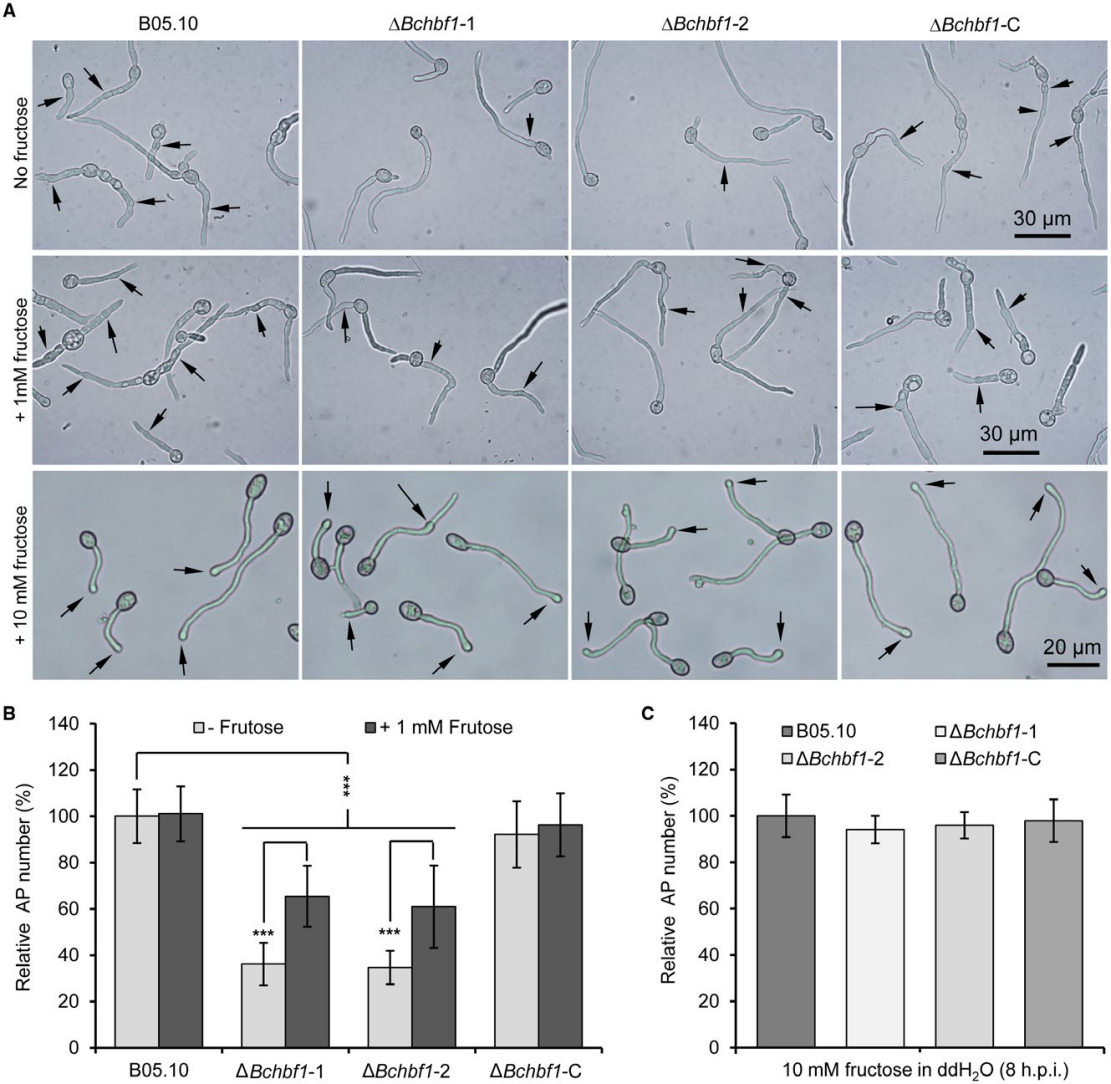
Loss of *BcHBF1 *reduces appressorium formation in *B. cinerea*. (A) *BcHBF1* is required for *B. cinerea* appressorium formation and addition of fructose promotes the structure formation in the ∆*Bchbf1 *mutant strains. (B and C) Quantification of appressorium formation in the absence or presence of low concentration (1 mM) of fructose (B) and of higher concentration (10 mM) of fructose (C). For each strain, more than 100 conidia were examined in each experiment. Data represent means ± standard deviations (SDs) from three independent experiments. ***: significant at *P* < 0.001.

**Figure 7:**
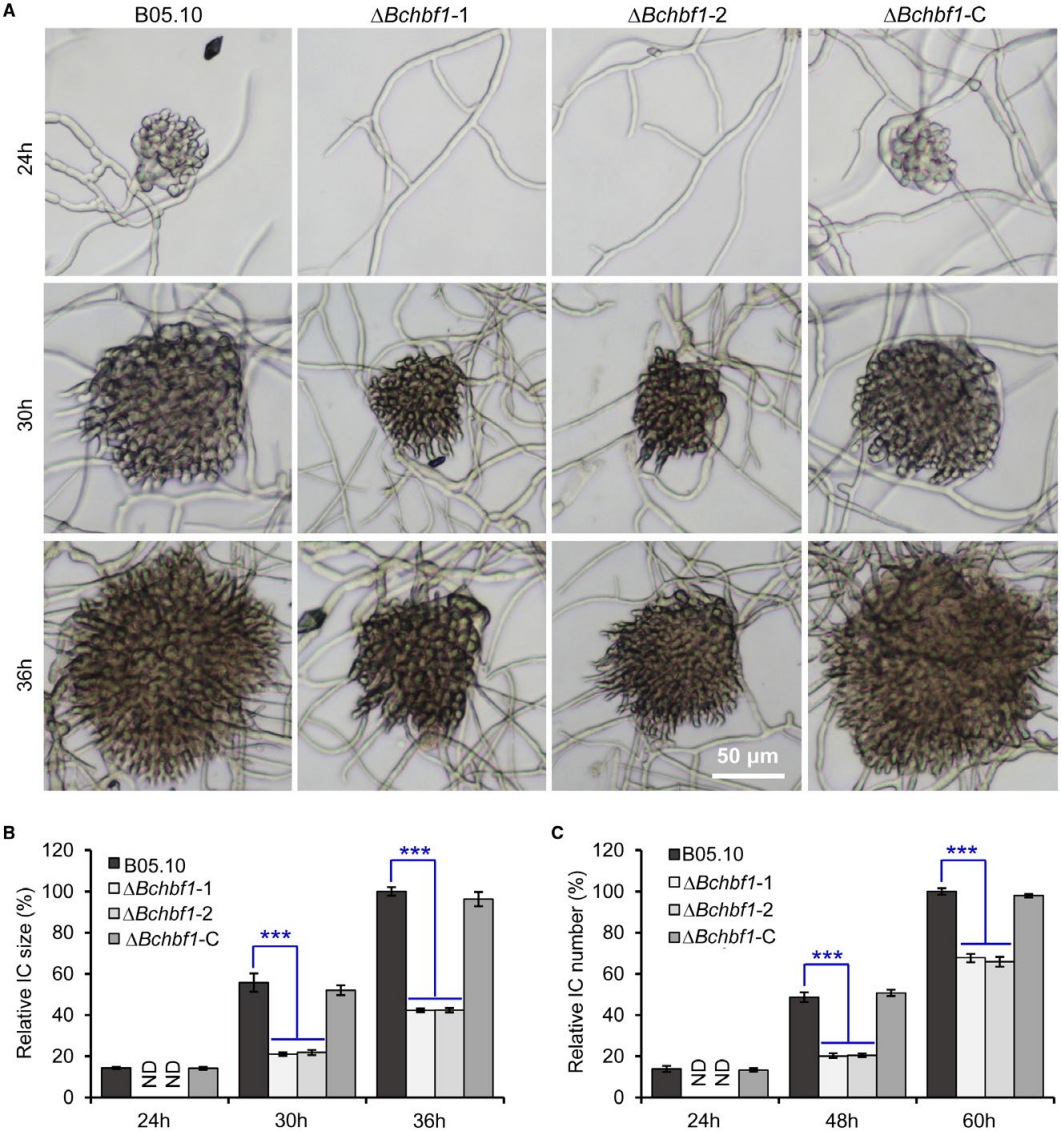
Loss of *BcHBF1* impairs infection cushion development in *B. cinerea*. (A) Infection cushion formation of the indicated *B. cinerea* WT, ∆*Bchbf1* and ∆*Bchbf1*‐C strains during a time course (36 h) of incubation at 20 ℃. Representative images are from one of three independent experiments; all the experiments resulted in similar results. (B and C) Quantification of the sizes (B) and numbers (C) of infection cushions produced by the indicated strains at the indicated hpi. Data represent means ± standard deviations (SDs) from three independent experiments in which triplicate slides were analyzed for each strain in each experiment. ***: significant at *P* < 0.001.

Infection structures play a critical role in *B. cinerea* host penetration and virulence (Cao *et al*., 2018; Feng *et al*., 2017; Liu *et al*., 2018). To evaluate the effect of *BcHBF1 *on infection structure formation, we determined appressorium and infection cushion formation of the WT, ∆*Bchbf1* and ∆*Bchbf1*‐C strains. Our data demonstrated that when the strains were incubated in ½ PDB on glass slides for 8 h, formation of appressoria by the mutant strains was significantly impaired; the relative appressorium production of the Δ*Bchbf1 *mutant strains was about 35% of the WT control and complemented strains (Fig. 6A and B). Appressorium formation in *B. cinerea* can be induced by fructose (Doehlemann *et al*., 2006; Liu *et al*., 2018), thus, we observed appressorium formation by conidia of the tested strains in the presence of different concentrations of fructose for 8 h. Our findings indicated that addition of fructose significantly (*P* < 0.001) facilitated appressorium formation by the Δ*Bchbf1 *mutant strains. In absence of fructose, only 35% of the mutant conidia formed the appressorium structures, whereas, in the presence of 1 mM fructose, about 60% of the mutant conidia formed the structure (Fig. 6A and B). Addition of fructose only slightly promoted appressorium formation by the WT and complemented strains; about 88% and 89%, 81% and 85% of the WT and complemented conidia formed appressoria in the absence and presence of fructose (1 mM) in ½ PDB, respectively (Fig. 6A and B). In the presence of 10 mM fructose, appressorium production by all the tested strains did not display significant difference (almost all the conidia produced appressorium structure) (Fig. 6A and C). When the tested strains were cultured with liquid CM on glass slides, the WT and complemented ∆*Bchbf1*‐C strains formed infection cushions at about 18 hpi and some of them were well developed at 24 hpi (Fig. 7A); formation of infection cushions by the ∆*Bchbf1* mutants was not observed until 30 hpi. At 36 hpi, infection cushions formed by the mutants were much smaller; the relative size of infection cushions produced by the mutants were only 42% of those of the WT and complemented strains (Fig. 7A and B). The numbers of infection cushions produced by the Δ*Bchbf1* mutants significantly reduced at 48 hpi and the numbers of infection cushions formed by the all the tested strains were gradually closer after 60 hpi (Fig. 7C). Taken together, these data demonstrated that *BcHBF1* plays a role in mediating infection structure formation, which may partially account for the virulence‐attenuation of the pathogen losing *BcHBF1*.

### *BcHBF1* is required for full virulence in *B. cinerea*

**Figure 8:**
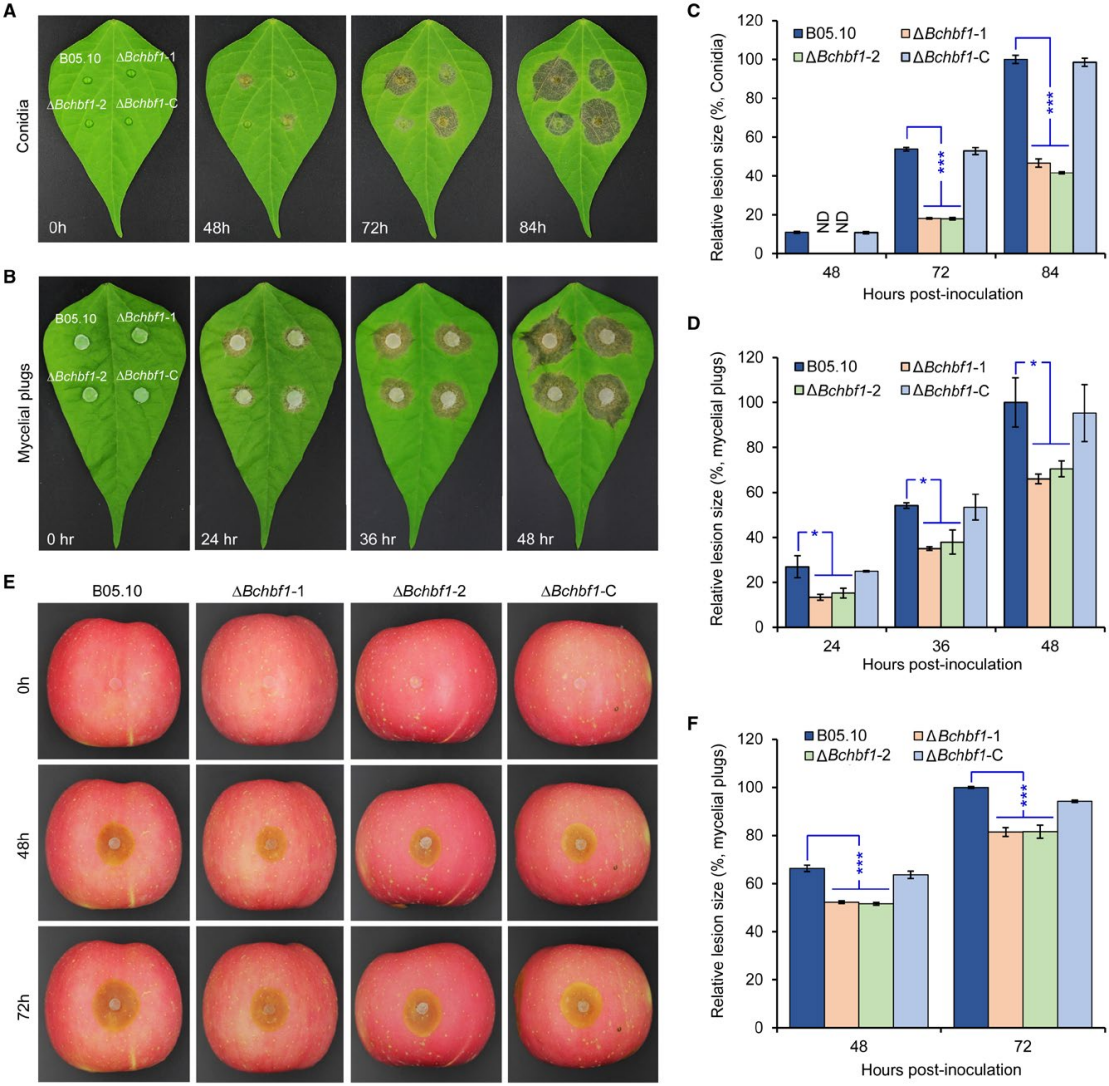
*BcHBF1* is required for full virulence of *B. cinerea. *(A, B) Diseased green bean leaves caused by the indicated *B. cinerea* WT, ∆*Bchbf1* and complemented strains during a time course (84 h) of infection. Droplets of conidial suspension (mixture of conidial suspension [1 × 10^6^ conidia/mL] and PDB, vol: vol = 1: 1, 5 µL) (A) and mycelial plugs (5 mm in diameter) (B) of each strain were inoculated. The inoculated leaves were incubated at 20 °C in dark and diseased leaves were photographically documented at the indicated hpi. (C and D) Quantification of the lesion sizes caused by the indicated strains shown in (A) and (B), respectively. (E) Loss of *BcHBF1 *in *B. cinerea* impairs virulence of the pathogen on apple fruit via wound‐inoculation approach. (F) Quantification of the lesion sizes caused by the indicated strains shown in (E). Representative images are from one experiment. Data represent means ± standard deviations (SDs) from at least four independent experiments. *, ***: significant at *P* < 0.05, 0.001, respectively.

To comprehensively evaluate the role of *BcHBF1* in *B. cinerea* virulence, we inoculated detached host leaves (green bean) with conidial suspension droplets (5 µL, 5 × 10^5^ conidia/mL) (Fig. 8A) and mycelial plugs (Fig. 8B). Our data demonstrated that in conidial suspension inoculation, lesions caused by the WT and complemented strains were observed at 48 hpi; the Δ*Bchbf1* mutant strains did not produce obvious lesions on green bean leaves until 72 hpi (Fig. 8A and C). In mycelial plug inoculation, lesions caused by all the tested strains were observed at 24 hpi; the Δ*Bchbf1* mutant strains also induced smaller lesions (*P* < 0.05) on green bean leaves at the indicated time points post‐inoculation (Fig. 8B and D). The results demonstrate that compared to the WT and Δ*Bchbf1‐*C strains, the grey mould disease caused by the mutants was less severe and delayed.

To investigate a role of *BcHBF1* on *B. cinerea* expansion *in planta*, we inoculated apple fruits with mycelial plugs via wound‐inoculation approach, which provides entrances for mycelia or germ tubes, thereby enabling pathogen entry into host cells via an infection structure‐independent mechanism. Our finding indicated that the Δ*Bchbf1* mutants could cause necrotic lesions. However, the relative lesion size induced by the mutants on wounded apple fruits was still significantly smaller than those induced by the WT and complemented strains (Fig. 8E and F). At 48 hpi and 72 hpi, the relative lesion size caused by the mutants reached 79% and 82%, respectively, of that caused by the WT control (Fig. 8E and F). These data suggest that *BcHBF1* plays an important role in *B. cinerea* invasive growth *in planta*.

**Figure 9:**
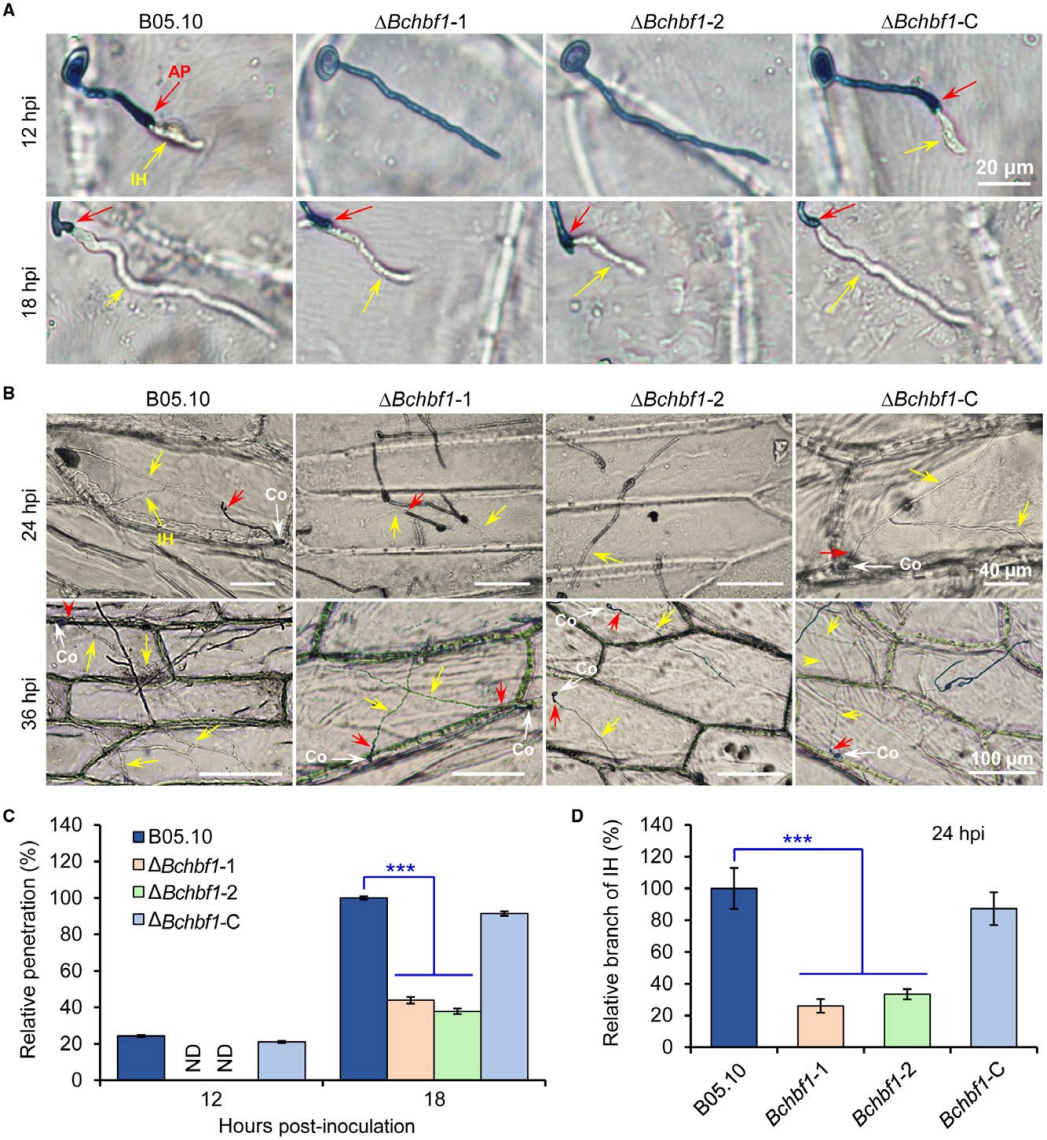
Loss of *BcHBF1* impairs *B. cinerea *host penetration and invasive hyphal development*.* (A) Loss of *BcHBF1* delays plant‐tissue penetration by the ∆*Bchbf1* mutants. Droplets (10 µL) of conidial suspension (mixture of conidial suspension [1 × 10^5^ conidia/mL] and ddH_2_O, vol: vol = 1: 10) of each strain were inoculated on onion epidermis (with extensive wash). The inoculated epidermis were incubated at 20 °C in dark; and at the indicated time points post‐inoculation, the inoculated epidermis were performed lactophenol blue staining and then photographically documented. (B) Disruption of *BcHBF1* impairs *B. cinerea* invasive hyphal *in planta* branching and growth during infection. Bars in upper and lower panels are 40 µm and 100 µm, respectively. (C) Quantification of host penetration by the indicated strains at 12 hpi and 18 hpi. (D) Quantification of branch number of invasive hyphae of the indicated strains at 24 hpi. Representative images are from one of three independent experiments. Red arrows: appressoria and/or penetration points. Yellow arrows: branched or unbranched invasive hyphae. Co: conidium. IH: invasive hyphae. Data represent means ± standard deviations (SDs) from at least three independent experiments. ***: significant at *P* < 0.001.

To test the effect of *BcHBF1 *on host invasion, we performed onion epidermal cell infection assay to analyse host penetration by the WT, Δ*Bchbf1* and complemented strains and found that at 12 hpi, the WT and complemented strains penetrated into onion epidermal cells and such penetration by the mutants was not observed until 18 hpi. However, the length of primary invasive hyphae of the WT and complemented strains was longer than that of the mutants (Fig. 9A). At 24 hpi to 36 hpi, compared to the WT and complemented strains, the mutants displayed fewer branch numbers (*P* < 0.001) of invasive hyphae (Fig. 9B and D) and less degree of invasive hyphal elongation (Fig. 9B). These findings suggest that it takes a longer time for the mutants to establish a successful host invasion and infection. Taken together, our data demonstrate that *BcHBF1* plays an important role in *B. cinerea* host penetration, invasive hyphal development *in planta*, and is required for the full virulence of the pathogens.

## Discussion

Understanding the molecular mechanisms of *B. cinerea* pathogenesis and host responses is critical for control of plant grey mould; and virulence‐associated factors that facilitate host infection by the aggressive pathogen play important roles in these processes. Insertional mutagenesis via the *Agrobacterium tumefaciens*‐mediated transformation (ATMT) method is a powerful approach to identify factors with novel functions in the fungal pathogen (Giesbert *et al*., 2012; Schumacher *et al*., 2014, 2018; Sharma *et al*., 2018). Using the ATMT approach, we previously identified novel pathogenesis‐associated factors, including the pre‐rRNA processing factor Nop53 (Cao *et al*., 2018) and phosphoenolpyruvate carboxykinase gene *BcPCK1* in gluconeogenesis (Liu *et al*., 2018) in *B. cinerea*, and dissected the mechanisms of how those factors mediate the vegetative and pathogenic development as well as virulence of the pathogen (Cao *et al*., 2018; Liu *et al*., 2018). In this study, we identified a virulence‐attenuated T‐DNA tagged mutant M8008 from the same *B. cinerea* T‐DNA mutant library. Further sequence, genetics and functional analyses demonstrate that *BcHBF1* is responsible for pathogenicity reduction in the mutant. *BcHBF1* is a novel *B. cinerea‐*specific factor whose homologs have not been identified in genomes of any other known organisms. *BcHBF1* mediates conidial morphogenesis, hyphal branching (both *in vitro* and *in planta*), infection structure development and virulence of the pathogen, which corresponds to the up‐regulation of *BcHBF1* expression during these processes. However, *BcHBF1 *plays a limited role in *B. cinerea* conidiation, conidial germination, radial growth of mycelia, osmotic‐ and oxidative‐stress adaptation as well as cell wall integrity.

Sclerotia serve as specific structures that survive in hostile environments including winters and grow out into hyphae under suitable conditions; they therefore act as the primary inoculum in the disease cycle. Moreover, sclerotia play an important role in sexual reproduction, in which *B. cinerea* sclerotia may serve as female (sclerotial) parental tissue, which can be fertilized by microconidia from an isolate of opposite mating type (the spermatial parent) to produce fruiting bodies that contain sexual ascospores (Faretra *et al*., 1988; Veloso and van Kan, 2018; Williamson *et al*., 2007). Our findings demonstrate that *BcHBF1* plays a crucial role in sclerotial formation in *B. cinerea*. Disruption of *BcHBF1 *dramatically delayed and reduced sclerotial formation as well as altered sclerotial morphology of the mutants (Fig. 4), implying that *BcHBF1 *may also regulate sexual reproduction of the pathogen via mediating sclerotial formation. In *B. cinerea* sexual production, the cellular details of plasmogamy and initiation of apothecia remain largely unknown. Moreover, the apothecia are undocumented or rare in most crops attacked by the pathogen (Williamson *et al*., 2007). Although our data indicated that loss of *BcHBF1* did not impair germination of the mutant sclerotia, the effect of *BcHBF1* on sexual production via sclerotial spermatization remains to be clarified. Many *B. cinerea* factors that are critical for *B. cinerea* sclerotial formation have been recently characterized, including diacylglycerol O‐acyl transferase 2 (DGAT2) (Sharma *et al*., 2018), autophagy‐related proteins (Liu *et al*., 2019; Ren *et al*., 2017, 2018a), the kynurenine 3‐monooxygenase (Kang *et al*., 2018), the essential ER protein BcPdi1 (Marschall and Tudzynski, 2017), and the MADS‐Box transcription factor Bcmads1 (Zhang *et al*., 2016). BcHbf1, together with these above‐mentioned factors, share a common in controlling the pathogen sclerotial formation; however, the association of these factors that are critical for the production of sclerotia remains obscure, and the clarification of the connection of these crucial factors for sclerotial production may constitute an intriguing research direction in the pathogen development.

Hyphal branching is frequently associated with pathogenicity of fungal pathogens. A successful host infection by fungal plant pathogens requires differential hyphal branching in the time course of infection (Liu *et al*., 2017; Mohammadi *et al*., 2017; Rolke and Tudzynski, 2008). The rice blast fungus *Magnaporthe oryzae* forms hyphae that do not branch prior to the penetration, and upon reaching epidermal cells, the hyphae of the pathogen start to branch, which enables extensive cell colonization (Caracuel‐Rios and Talbot, 2007). During infection, the plant pathogen *Claviceps purpurea*, which causes ergot of cereals and grasses, forms hyphae with extreme apical dominance; however, after tapping the vascular bundles, the fungus hyphae undergo frequent branching required for colonization of the whole ovary (Rolke and Tudzynski, 2008). The ZtVf1 transcription factor in wheat pathogen *Zymoseptoria tritici* is required for the full virulence and involved in hyphal branching of the pathogen. Loss of ZtVf1 reduces hyphal branching and biomass production of the *ZtVf1* mutant. The reduced virulence and tissue colonization of the *ZtVf1* mutant might be partly attributed to the lower hyphal branching and less fungal biomass production (Mohammadi *et al*., 2017). In this study, we demonstrate that loss of *BcHBF1* resulted in a lower degree of hyphal branching in both vegetative growth and host infection, suggesting that the reduced virulence of the ∆*Bchbf1* mutant might be partly attributed to a lower degree of invasive hyphal branching and elongation or development.

*B. cinerea* penetrates into host plant cells or tissues mainly via its infection structures that include appressoria and infection cushions (Choquer *et al*., 2007; Van den Heuvel and Waterreus, 1983). Disruption of *BcHBF1 *reduced appressorium formation by the mutant strains (Fig. 6A and B) and delayed host penetration by the mutant appressoria (Fig. 9A and C). Loss of *BcHBF1 *greatly delays and reduces infection structure formation by the mutants (Figs. 6 and 7). Pathogenicity assays for the ∆*Bchbf1 *mutants using both the mutant conidia and mycelial plugs demonstrate that loss of *BcHBF1 *in the pathogen significantly impaired virulence of the mutants (Fig. 8), which corresponds to the reduction in appressorium formation, the delay of infection cushion formation and appressorium host penetration. The mutant strains displayed similar osmotic‐ and oxidative‐stress adaptation as well as cell wall integrity, implying that the adaptation of the ∆*Bchbf1 *mutants to the *in planta* stresses, if any, imposed by hosts upon penetration into host cells, is not impaired. These findings suggest that the reduced mutant appressoria as well as the delay of appressorium host penetration and infection cushion formation by the ∆*Bchbf1 *mutants may mainly account for their virulence reduction.

The mechanism of *BcHBF1* mediating *B. cinerea *development and virulence remains to be characterized. Although the ∆*Bchbf1 *mutant strains could form a small number of appressoria, the delay of host penetration by the mutant appressoria was observed (Fig. 9A); this may partly account for the attenuated pathogenicity of the mutant strains, and raises an intriguing question about how *BcHBF1* influences the capability of appressorium penetration. Analysis of *BcHBF1* expression profiles demonstrates that *BcHBF1* expression was up‐regulated during the development of young mycelia and host infection, suggesting that *BcHBF1* is required for the pathogen germ tube and hyphal development as well as host infection. The expression profiles of *BcHBF1* also correspond to the phenotypic defects including hyphal development and branching, appressorium and infection cushion formation, host penetration and invasive hyphal development of the *BcHBF1 *deletion mutants in these processes. However, the signal that stimulates the up‐regulation of the gene remains unknown. Subcellular localization is a key functional characteristic of a protein; therefore, subcellular localization of BcHbf1 needs to be determined, although bioinformatics predicts that BcHbf1 may be a transmembrane protein (Fig. S1B). Bioinformatics analysis suggests that the known functional domains of the deduced BcHbf1 are not detected, and functional characteristics of the protein also need to be further investigated. All the above‐mentioned issues may constitute a research direction to reveal the mysterious veil of this *B. cinerea*‐specific factor.

In summary, we identified a novel *B. cinerea*‐specific factor *BcHBF1* from a *B. cinerea* T‐DNA mutant library and demonstrated that the *B. cinerea‐*specific factor plays important roles in the fungus pathogenic development and virulence. Our work provides new insights into fungal factors that enhance virulence of the grey mould fungus via promoting its infection structure formation, host penetration and invasive hyphal branching and growth.

## Experimental Procedures

### Fungal strains and culture conditions

*B. cinerea* WT (B05.10) and its derived strains including *BcHBF1* gene deletion mutants ∆*Bchbf1‐*1, ∆*Bchbf1‐*2, and the ∆*Bchbf1‐*1 complemented (∆*Bchbf1‐*C) strains were cultivated and maintained on potato dextrose agar (PDA) or CM as previously described (Liu *et al*., 2018).

### Identification of pathogenicity‐associated gene *BcHBF1*

A *B. cinerea* T‐DNA tagged transformant library (containing ~50 000 transformants) in the WT strain B05.10 was generated in our laboratory by using the ATMT approach as previously described (Giesbert *et al*., 2012; Rolland *et al*., 2003). A pathogenicity‐attenuated mutant strain M8008 was identified from the library via screening for virulence‐attenuated mutants by using both detached tomato and strawberry leaves as hosts. T‐DNA insertion regions were analysed by using TAIL‐PCR analysis method (Liu and Whittier, 1995; Terauchi and Kahl, 2000). Analyses of the T‐DNA flanking sequences and the deduced amino acid sequences of homologous genes were performed as previously described (Cao *et al*., 2018; Liu *et al*., 2018).

### Generation of gene deletion mutants and complemented strains

Generation of *BcHBF1 *deletion mutant (∆*Bchbf1*) and the genetic complemented strain ∆*Bchbf1‐*C were performed with previously described approaches (Cao *et al*., 2018; Feng *et al*., 2017; Liu *et al*., 2018). Briefly, vector pXEH containing hygromycin phosphotransferase gene (*HPH*) cassette was used for targeted gene replacement. The 5’‐ and 3’‐homologous flanks of the targeted gene were amplified and cloned into pXEH vector in the upstream and downstream of *HPH*, respectively. The gene KO vector was transformed into *A. tumefaciens* strain AGL‐1 as previously described (Feng *et al*., 2017). The resultant deletion transformants were screened on PDA with 100 μg/mL hygromycin.

Vector pXEBA conferring glufosinate‐ammonium resistance was used for complementation of the ∆*Bchbf1 *mutants. To generate complemented vector, a fragment containing 1591 bp upstream and 577 bp downstream of the coding region of *BcHBF1* was amplified by PCR and cloned into vector pXEBA. The complementary vector was transformed into *A. tumefaciens* strain AGL‐1 and the resultant transformants generated by the ATMT method were screened on glufosinate‐ammonium containing DCM medium (Liu *et al*., 2018). Diagnostic PCR was performed to verify the integration events of the selected transformants. The gene deletion and complemented strains were further confirmed by qRT‐PCR (Liu *et al*., 2018; Weiberg *et al*., 2013). Primers used in those experiments are listed in Table S1.

### Fungal developmental assays

Fungal growth of the tested *B. cinerea* strains was determined by measuring the radial diameter of colonies on solid CM (1% glucose, 0.2% peptone, 0.1% yeast extract, 0.1% casamino acids, nitrate salts, trace elements, 0.01% vitamins, 1.2% agar, pH 6.5). Other media used in the assays included liquid CM (CM without agar), PDA (200 g potato, 20 g glucose, 20 g agar and 1 L water), and PDB (200 g potato, 20 g glucose and 1 L water).

Fungal conidiation, conidial morphology, conidium germination, infection structure formation and sclerotial formation were determined as previously described (Feng *et al*., 2017). Briefly, for conidium germination assays, fresh conidia of the WT, ∆*Bchbf1* and ∆*Bchbf1*‐C strains were harvested from PDA or CM plates with ddH_2_O and the conidial suspension was adjusted to the concentration of 5 × 10^5 ^conidia/mL in ½ PDB. For infection cushion formation assays, droplets (20 μL) of liquid CM were placed on the surfaces of the glass slides, then conidial suspension droplets (5 × 10^5^ conidia/mL, 2 μL) were added to the liquid media and quickly mixed, the inoculated slides were incubated in a moistened chamber at 20 °C. At the indicated time points post‐incubation, formation of infection cushions was observed and photographically documented. The abilities of infection cushion formations were determined by microscopic examination of the total number of infection cushions in five randomly selected view areas per replicate. Observation of conidial germination, infection structure formation, etc. was performed with a Nikon Eclipse 80i fluorescence microscope system. For sclerotial formation assays, strains were cultivated on CM plates at 20 °C in darkness; production of sclerotia by the test strains was observed and photographically documented during a time course of 30 days of incubation. At least three independent experiments with triplicated replicates per experiment were performed. Sclerotial germination assays were performed as previously described (Liu *et al*., 2018).

### Plant infection experiments

Conidia of *B. cinerea* WT, ∆*Bchbf1* and complemented strains cultivated on PDA at 20°C for 10 days to 15 days were collected, washed, and prepared to spore suspension with a concentration of 1 × 10^6^ conidia/mL. For conidial suspension inoculation, the conidia were suspended in liquid CM. Droplets of 5 μL of spore suspension with 5 × 10^5^ conidia/mL were inoculated on 3‐ to 6‐week‐old detached green bean leaves. For mycelial plug inoculation, green bean leaves and/or apple fruits were inoculated with actively growing *B. cinerea* mycelial plugs (5 mm in diameter) taken from a 3‐ to 4‐day‐old culture of the tested strains. Mycelial plug and conidial inoculated materials were incubated in containers with a sheet of plastic film sealed on the top of each container to maintain a high humidity of infection condition. The inoculated materials were photographically documented at 0, 24, 36, 48, 72, 84 and 96 hpi.

### Stress adaptation assays

The adaptation of the tested strains to different stresses was analysed as previously described (Cao *et al*., 2018; Feng *et al*., 2017; Liu *et al*., 2018). Briefly, conidial suspension (1 μL, 1 × 10^6 ^conidia/mL) or mycelial plugs (5 mm in diameter) of the *B. cinerea* WT, ∆*Bchbf1* and complemented strains were inoculated onto CM supplemented with different concentrations of stress agents, including osmotic stress agents NaCl and KCl, oxidative‐stress reagent H_2_O_2_ and cell wall disturbing agents SDS and CR. The inoculated plates were incubated in incubators at 20  °C. The colony diameters of the strains were measured at the indicated hpi and stress adaptation of the strains was determined by their mycelial growth and growth inhibition (Jiang *et al*., 2012). Triplicate colonies for each strain were analysed in each experiment and at least three independent experiments were performed.

### Cytological assay

The preparation of conidia and onion epidermal cells and sample lactophenol blue staining were performed as previously described (Liu *et al*., 2018). The infected samples were microscopically observed, photographically documented and analysed at 12, 18, 24 and 36 hpi.

### Quantitative Reverse Transcription‐Polymerase Chain Reaction (**qRT‐PCR)**

For conidial germination, spore suspension (100 μL) of each tested strain was plated on cellophane placed on the top of PDA plates, at 0, 1, 2, 4, 6, 8, 12 and 24 hpi; germinating conidia were collected for RNA extraction. For host infection, spore suspension (2 × 10^6 ^conidia/mL) of each strain was first diluted with liquid CM (1: 1, vol: vol) and then droplets (50 μL) of the diluted conidial suspension of each strain were inoculated on the intact leaves of green bean and incubated at room temperature. At 0, 6, 12, 24, 36 and 48 hpi, inoculated leaf samples were collected for RNA extraction. Total RNA was extracted and purified using RNase‐free DNase I to remove genomic DNA (TaKaRa, Dalian, China), and 1 μg of RNA sample of each strain was used for the first strand cDNA synthesis with the PrimeScript® RT Reagent Kit (TaKaRa, Dalian, China). Real‐time PCR was conducted using SYBR^®^ Green I fluorescent dye detection (TaKaRa, Dalian, China). The pathogen actin gene *BcACT1 *was used as endogenous reference to normalize the expression levels of the measured genes, and the relative expression levels of the target genes were analysed using the relative 2^–ΔΔCt^ method (Livak and Schmittgen, 2001).

### Statistical analysis

The quantitative data presented in this study were derived from at least three independent experiments with triplicate treatments examined. To make the results from different independent experiments comparable, the data of controls including mycelial growth, lesion size, conidial germination, etc. in each independent experiment were normalized as 100%. The significance of the data was assessed using the Student’s *t*‐test. A *P*‐value of < 0.05 was considered as a significant difference.

## Author contributions

Q‐MQ, J‐KL and YL conceived the experiments; YL, J‐KL, JH, JS, M‐ZZ, Y‐YZ and Y‐YW, performed the experiments; Q‐MQ, YL and J‐KL analysed and interpreted the data; Q‐MQ and G‐HL provided reagents, Q‐MQ supervised the work; Q‐MQ and J‐KL wrote the paper.

## Competing interests

The authors declare that no competing interests exist.

## Supporting information


**Fig. S1** Sequence analysis of *BcHBF1* in B. cinerea. (A) cDNA and deduced amino acid sequence of *BcHBF1*. (B) The deduced protein domains and functional sites of BcHbf1 based on InterProScan (http://www.ebi.ac.uk/interpro/scan.html) analyses.

**Fig. S2** *BcHBF1* is dispensable for *B. cinerea* mycelial growth. (A and B) *BcHBF1 *mediation of radial growth of mycelia (A) and conidial development (B) of the indicated *B. cinerea* strains on complete medium (CM) plates at 20 °C. (C and D) Quantification of radical growth of mycelia (C) and conidial development (D) of the indicated strains cultured on CM for 3 days and 4 days, respectively. Data represent means ± SD from three independent experiments with triplicate plates examined for each treatment. *, **, ***: significance at *P* < 0.05, *P *< 0.01 and *P *< 0.001, respectively.

**Fig. S3** *BcHBF1* is required for hyphal branching. (A) Droplets of conidial suspension (mixture of conidial suspension [1 × 10^5^ conidia mL for the time point 32 hpi, 1 × 10^6^ conidia mL for other time points] and PDB, vol: vol   1:1, 10 µl) of each strain were inoculated on glass slides and incubated at 20 °C. Germinated conidia were photographically documented at the indicated hpi. (B and C) Quantification of hyphal branching (B) and length (C) of the indicated strains at the indicated hpi. Data represent means ± SD from at least three independent experiments with triplicate slides examined for each treatment. *, **, ***: significance at *P *< 0.05, *P *< 0.01 and *P* < 0.001, respectively.

**Table S1** Primers used in this study.

## References

[mpp12788-bib-0001] Amselem, J. , Cuomo, C.A. , van Kan, J.A.L. , Viaud, M. , Benito, E.P. , Couloux, A. , Coutinho, P.M. , de Vries, R.P. , Dyer, P.S. , Fillinger, S. , Fournier, E. , Gout, L. , Hahn, M. , Kohn, L. , Lapalu, N. , Plummer, K.M. , Pradier, J.‐M. , Quévillon, E. , Sharon, A. , Simon, A. , ten Have, A. , Tudzynski, B. , Tudzynski, P. , Wincker, P. , Andrew, M. , Anthouard, V. , Beever, R.E. , Beffa, R. , Benoit, I. , Bouzid, O. , Brault, B. , Chen, Z. , Choquer, M. , Collémare, J. , Cotton, P. , Danchin, E.G. , Da Silva, C. , Gautier, A. , Giraud, C. , Giraud, T. , Gonzalez, C. , Grossetete, S. , Güldener, U. , Henrissat, B. , Howlett, B.J. , Kodira, C. , Kretschmer, M. , Lappartient, A. , Leroch, M. , Levis, C. , Mauceli, E. , Neuvéglise, C. , Oeser, B. , Pearson, M. , Poulain, J. , Poussereau, N. , Quesneville, H. , Rascle, C. , Schumacher, J. , Ségurens, B. , Sexton, A. , Silva, E. , Sirven, C. , Soanes, D.M. , Talbot, N.J. , Templeton, M. , Yandava, C. , Yarden, O. , Zeng, Q. , Rollins, J.A. , Lebrun, M.‐H. and Dickman, M . (2011) Genomic analysis of the necrotrophic fungal pathogens *Sclerotinia sclerotiorum* and *Botrytis cinerea* . PLoS Genet. 7, e1002230. 21876677 10.1371/journal.pgen.1002230 PMC3158057

[mpp12788-bib-0002] Cao, S.N. , Yuan, Y. , Qin, Y.H. , Zhang, M.Z. , de Figueiredo, P. , Li, G.H. and Qin, Q.M. (2018) The pre‐rRNA processing factor Nop53 regulates fungal development and pathogenesis via mediating production of reactive oxygen species. Environ. Microbiol. 20, 1531–1549. 29488307 10.1111/1462-2920.14082

[mpp12788-bib-0003] Caracuel‐Rios, Z. and Talbot, N.J. (2007) Cellular differentiation and host invasion by the rice blast fungus *Magnaporthe grisea* . Curr. Opin. Microbiol. 10, 339–345. 17707684 10.1016/j.mib.2007.05.019

[mpp12788-bib-0004] Choquer, M. , Fournier, E. , Kunz, C. , Levis, C. , Pradier, J.‐M. , Simon, A. and Viaud, M. (2007) *Botrytis cinerea* virulence factors: new insights into a necrotrophic and polyphageous pathogen. FEMS Microbiol. Lett. 277, 1–10. 17986079 10.1111/j.1574-6968.2007.00930.x

[mpp12788-bib-0005] Dean, R. , Van Kan, J.A. , Pretorius, Z.A. , Hammond‐Kosack, K.E. , Di Pietro, A. , Spanu, P.D. , Rudd, J.J. , Dickman, M. , Kahmann, R. and Ellis, J. (2012) The Top 10 fungal pathogens in molecular plant pathology. Mol. Plant Pathol. 13, 414–430. 22471698 10.1111/j.1364-3703.2011.00783.x PMC6638784

[mpp12788-bib-0006] Doehlemann, G. , Berndt, P. and Hahn, M. (2006) Different signalling pathways involving a Gα protein, cAMP and a MAP kinase control germination of *Botrytis cinerea* conidia. Mol. Microbiol. 59, 821–835. 16420354 10.1111/j.1365-2958.2005.04991.x

[mpp12788-bib-0007] Faretra, F. , Antonacci, E. and Pollastro, S. (1988) Sexual behaviour and mating system of Botryotinia fuckeliana, teleomorph of *Botrytis cinerea* . Microbiol. 134, 2543–2550.

[mpp12788-bib-0008] Feng, H.Q. , Li, G.H. , Du, S.W. , Yang, S. , Li, X.Q. , de Figueiredo, P. and Qin, Q.M. (2017) The septin protein Sep4 facilitates host infection by plant fungal pathogens via mediating initiation of infection structure formation. Environ. Microbiol. 19, 1730–1749. 27878927 10.1111/1462-2920.13613

[mpp12788-bib-0009] Fillinger, S. and Elad, Y. (2016) Botrytis – the Fungus, the Pathogen and its Management in Agricultural Systems. Switzerland: Springer International Publishing.

[mpp12788-bib-0010] Frias, M. , Gonzalez, C. and Brito, N. (2011) BcSpl1, a cerato‐platanin family protein, contributes to *Botrytis cinerea* virulence and elicits the hypersensitive response in the host. New Phytol. 192, 483–495. 21707620 10.1111/j.1469-8137.2011.03802.x

[mpp12788-bib-0011] Giesbert, S. , Schumacher, J. , Kupas, V. , Espino, J. , Segmüller, N. , Haeuser‐Hahn, I. , Schreier, P. and Tudzynski, P. (2012) Identification of Pathogenesis‐Associated Genes by T‐DNA–Mediated Insertional Mutagenesis in *Botrytis cinerea*: A Type 2A Phosphoprotein Phosphatase and an SPT3 Transcription Factor Have Significant Impact on Virulence. Mol. Plant–Microbe Interact. 25, 481–495. 22112214 10.1094/MPMI-07-11-0199

[mpp12788-bib-0012] Gourgues, M. , Brunet‐Simon, A. , Lebrun, M.H. and Levis, C. (2004) The tetraspanin BcPls1 is required for appressorium‐mediated penetration of *Botrytis cinerea* into host plant leaves. Mol. Microbiol. 51, 619–629. 14731267 10.1046/j.1365-2958.2003.03866.x

[mpp12788-bib-0013] Hahn, M. (2014) The rising threat of fungicide resistance in plant pathogenic fungi: *Botrytis* as a case study. J. Chem. Biol. 7, 133–141. 25320647 10.1007/s12154-014-0113-1 PMC4182335

[mpp12788-bib-0014] Jiang, J. , Yun, Y. , Liu, Y. and Ma, Z. (2012) FgVELB is associated with vegetative differentiation, secondary metabolism and virulence in *Fusarium graminearum* . Fungal Genet. Biol. 49, 653–662. 22713714 10.1016/j.fgb.2012.06.005

[mpp12788-bib-0015] van Kan, J.A. (2006) Licensed to kill: the lifestyle of a necrotrophic plant pathogen. Trends Plant Sci. 11, 247–253. 16616579 10.1016/j.tplants.2006.03.005

[mpp12788-bib-0016] van Kan, J.A. , Shaw, M.W. and Grant‐Downton, R.T. (2014) *Botrytis species*: relentless necrotrophic thugs or endophytes gone rogue? Mol. Plant Pathol. 15, 957–961. 24754470 10.1111/mpp.12148 PMC6638755

[mpp12788-bib-0017] Kang, Z. , Yan, X. , Zang, J. , Wang, M. , Zhao, F. , Li, P. , Cao, H. , Han, J. , Xing, J. and Dong, J. (2018) The kynurenine 3‐monooxygenase encoding gene, BcKMO, is involved in the growth, development, and pathogenicity of *Botrytis cinerea* . Front. Microbiol. 9, 1039. doi:10.3389/fmicb.2018.01039. 29867912 PMC5968091

[mpp12788-bib-0018] Leroch, M. , Plesken, C. , Weber, R.W. , Kauff, F. , Scalliet, G. and Hahn, M. (2013) Gray mold populations in German strawberry fields are resistant to multiple fungicides and dominated by a novel clade closely related to *Botrytis cinerea* . Appl. Environ. Microbiol. 79, 159–167. 23087030 10.1128/AEM.02655-12 PMC3536109

[mpp12788-bib-0019] Liu, Y.G. and Whittier, R.F. (1995) Thermal asymmetric interlaced PCR: automatable amplification and sequencing of insert end fragments from P1 and YAC clones for chromosome walking. Genomics 25, 674–681. 7759102 10.1016/0888-7543(95)80010-j

[mpp12788-bib-0020] Liu, L. , Yan, Y. , Huang, J. , Hsiang, T. , Wei, Y. , Li, Y. , Gao, J. and Zheng, L. (2017) A Novel MFS Transporter Gene ChMfs1 Is Important for Hyphal Morphology, Conidiation, and Pathogenicity in *Colletotrichum higginsianum* . Front. Microbiol. 8, 1953. doi:10.3389/fmicb.2017.01953. 29067014 PMC5641377

[mpp12788-bib-0021] Liu, J.K. , Chang, H.W. , Liu, Y. , Qin, Y.H. , Ding, Y.H. , Wang, L. , Zhao, Y. , Zhang, M.Z. , Cao, S.N. , Li, L.T. and Liu, W. (2018) The key gluconeogenic gene PCK1 is crucial for virulence of *Botrytis cinerea* via initiating its conidial germination and host penetration. Environ. Microbiol. 20, 1794–1814. 29614212 10.1111/1462-2920.14112

[mpp12788-bib-0022] Liu, N. , Ren, W. , Li, F. , Chen, C. and Ma, Z. (2019). Involvement of the cysteine protease bcatg4 in development and virulence of botrytis cinerea. Curr. Genet. 65, 293–300. 30167777 10.1007/s00294-018-0882-0

[mpp12788-bib-0023] Livak, K.J. and Schmittgen, T.D. (2001) Analysis of relative gene expression data using real‐time quantitative PCR and the 2(‐Delta Delta C(T)) Method. Methods 25, 402–408. 11846609 10.1006/meth.2001.1262

[mpp12788-bib-0024] Marschall, R. and Tudzynski, P. (2016) Reactive oxygen species in development and infection processes. Semin. Cell Dev. Biol. 57, 138–146. 27039026 10.1016/j.semcdb.2016.03.020

[mpp12788-bib-0025] Marschall, R. and Tudzynski, P. (2017) The Protein Disulfide Isomerase of Botrytis cinerea: An ER Protein Involved in Protein Folding and Redox Homeostasis Influences NADPH Oxidase Signaling Processes. Front. Microbiol. 8, 960. doi:10.3389/fmicb.2017.00960. 28611757 PMC5447010

[mpp12788-bib-0026] Mohammadi, N. , Mehrabi, R. , Mirzadi Gohari, A. , Mohammadi Goltapeh, E. , Safaie, N. and Kema, G.H.J. (2017) The ZtVf1 transcription factor regulates development and virulence in the foliar wheat pathogen *Zymoseptoria tritici* . Fungal Genet. Biol. 109, 26–35. 29031630 10.1016/j.fgb.2017.10.003

[mpp12788-bib-0027] Nakajima, M. and Akutsu, K. (2014) Virulence factors of *Botrytis cinerea* . J. Gen. Plant Pathol. 80, 15–23.

[mpp12788-bib-0028] Ren, W. , Zhang, Z. , Shao, W. , Yang, Y. , Zhou, M. and Chen, C. (2017) The autophagy‐related gene BcATG1 is involved in fungal development and pathogenesis in *Botrytis cinerea* . Mol. Plant Pathol. 18, 238–248. 26972592 10.1111/mpp.12396 PMC6638273

[mpp12788-bib-0029] Ren, W. , Liu, N. , Sang, C. , Shi, D. , Zhou, M. , Chen, C. , Qin, Q. and Chen, W. (2018a) The autophagy gene BcATG8 regulates vegetative differentiation and plant infection of *Botrytis cinerea* . Appl. Environ. Micobiol. doi:10.1128/AEM.02455-17. PMC5960959 29572212

[mpp12788-bib-0030] Ren, W. , Sang, C. , Shi, D. , Song, X. , Zhou, M. and Chen, C. (2018b) Ubiquitin‐like activating enzymes BcAtg3 and BcAtg7 participate in development and pathogenesis of *Botrytis cinerea* . Curr. Genet. 64, 919–930. 29417220 10.1007/s00294-018-0810-3

[mpp12788-bib-0031] Rolke, Y. and Tudzynski, P. (2008) The small GTPase Rac and the p21‐activated kinase Cla4 in *Claviceps purpurea*: interaction and impact on polarity, development and pathogenicity. Mol. Microbiol. 68, 405–423. 18284596 10.1111/j.1365-2958.2008.06159.x

[mpp12788-bib-0032] Rolland, S. , Jobic, C. , Fevre, M. and Bruel, C. (2003) Agrobacterium‐mediated transformation of *Botrytis cinerea*, simple purification of monokaryotic transformants and rapid conidia‐based identification of the transfer‐DNA host genomic DNA flanking sequences. Curr. Genet. 44, 164–171. 12937946 10.1007/s00294-003-0438-8

[mpp12788-bib-0033] Romanazzi, G. , Smilanick, J.L. , Feliziani, E. and Droby, S. (2016) Integrated management of postharvest gray mold on fruit crops. Postharvest Biol. Technol. 113, 69–76.

[mpp12788-bib-0034] Schumacher, J. , Simon, A. , Cohrs, K.C. , Viaud, M. and Tudzynski, P. (2014) The transcription factor BcLTF1 regulates virulence and light responses in the necrotrophic plant pathogen *Botrytis cinerea* . PLoS Genet. 10, e1004040. 24415947 10.1371/journal.pgen.1004040 PMC3886904

[mpp12788-bib-0035] Schumacher, J. , Studt, L. and Tudzynski, P. (2018) The putative H3K36 demethylase BcKDM1 affects virulence, stress responses and photomorphogenesis in *Botrytis cinerea* . Fungal Genet. Biol. doi:10.1016/j.fgb.2018.11.003. 30445217

[mpp12788-bib-0036] Sharma, E. and Kapoor, R. (2017) Insights into the molecular interplay of virulence factors in *Botrytis cinerea* . Australas. Plant Pathol. 46, 551–561.

[mpp12788-bib-0037] Sharma, E. , Tayal, P. , Anand, G. , Mathur, P. and Kapoor, R. (2018) Functional analysis of diacylglycerol O‐acyl transferase 2 gene to decipher its role in virulence of *Botrytis cinerea* . Curr. Genet. 64, 443–457. 28940057 10.1007/s00294-017-0752-1

[mpp12788-bib-0038] Terauchi, R. and Kahl, G. (2000) Rapid isolation of promoter sequences by TAIL‐PCR: the 5'‐flanking regions of Pal and Pgi genes from yams (Dioscorea). Mol. Gen. Genet. 263, 554–560. 10821191 10.1007/s004380051201

[mpp12788-bib-0039] Van den Heuvel, J. and Waterreus, L.P. (1983) Conidial concentration as an important factor determining the type of prepenetration structures formed by *Botrytis cinerea* on leaves of French bean (Phaseolus vulgaris). Plant Pathol. 32, 263–272.

[mpp12788-bib-0040] Van Kan, J.A. , Stassen, J.H. , Mosbach, A. , Van Der Lee, T.A. , Faino, L. , Farmer, A.D. , Papasotiriou, D.G. , Zhou, S. , Seidl, M.F. and Cottam, E. (2017) A gapless genome sequence of the fungus *Botrytis cinerea* . Mol. Plant Pathol. 18, 75–89. 26913498 10.1111/mpp.12384 PMC6638203

[mpp12788-bib-0041] Veloso, J. and van Kan, J.A.L. (2018) Many Shades of Grey in *Botrytis*‐Host Plant Interactions. Trends Plant Sci. 23, 613–622. 29724660 10.1016/j.tplants.2018.03.016

[mpp12788-bib-0042] Wang, M. , Weiberg, A. , Dellota, E. Jr , Yamane, D. and Jin, H. (2017) *Botrytis* small RNA Bc‐siR37 suppresses plant defense genes by cross‐kingdom RNAi. RNA Biol. 14, 421–428. 28267415 10.1080/15476286.2017.1291112 PMC5411126

[mpp12788-bib-0043] Weiberg, A. , Wang, M. , Lin, F.M. , Zhao, H. , Zhang, Z. , Kaloshian, I. , Huang, H.D. and Jin, H. (2013) Fungal small RNAs suppress plant immunity by hijacking host RNA interference pathways. Science 342, 118–123. 24092744 10.1126/science.1239705 PMC4096153

[mpp12788-bib-0044] Williamson, B. , Tudzynski, B. , Tudzynski, P. and van Kan, J.A. (2007) *Botrytis cinerea*: the cause of grey mould disease. Mol. Plant Pathol. 8, 561–580. 20507522 10.1111/j.1364-3703.2007.00417.x

[mpp12788-bib-0045] Zhang, Z. , Li, H. , Qin, G. , He, C. , Li, B. and Tian, S. (2016) The MADS‐Box transcription factor Bcmads1 is required for growth, sclerotia production and pathogenicity of *Botrytis cinerea* . Sci. Rep. 6, 33901. 27658442 10.1038/srep33901 PMC5034256

