# Supplementary figures and images for: A novel Botrytis cinerea‐specific gene BcHBF1 enhances virulence of the grey mould fungus via promoting host penetration and invasive hyphal development
Source: Mol Plant Pathol. 2019 Apr 22;20(5):731–47. doi: 10.1111/mpp.12788 (PMC6637910; doi:10.1111/mpp.12788)

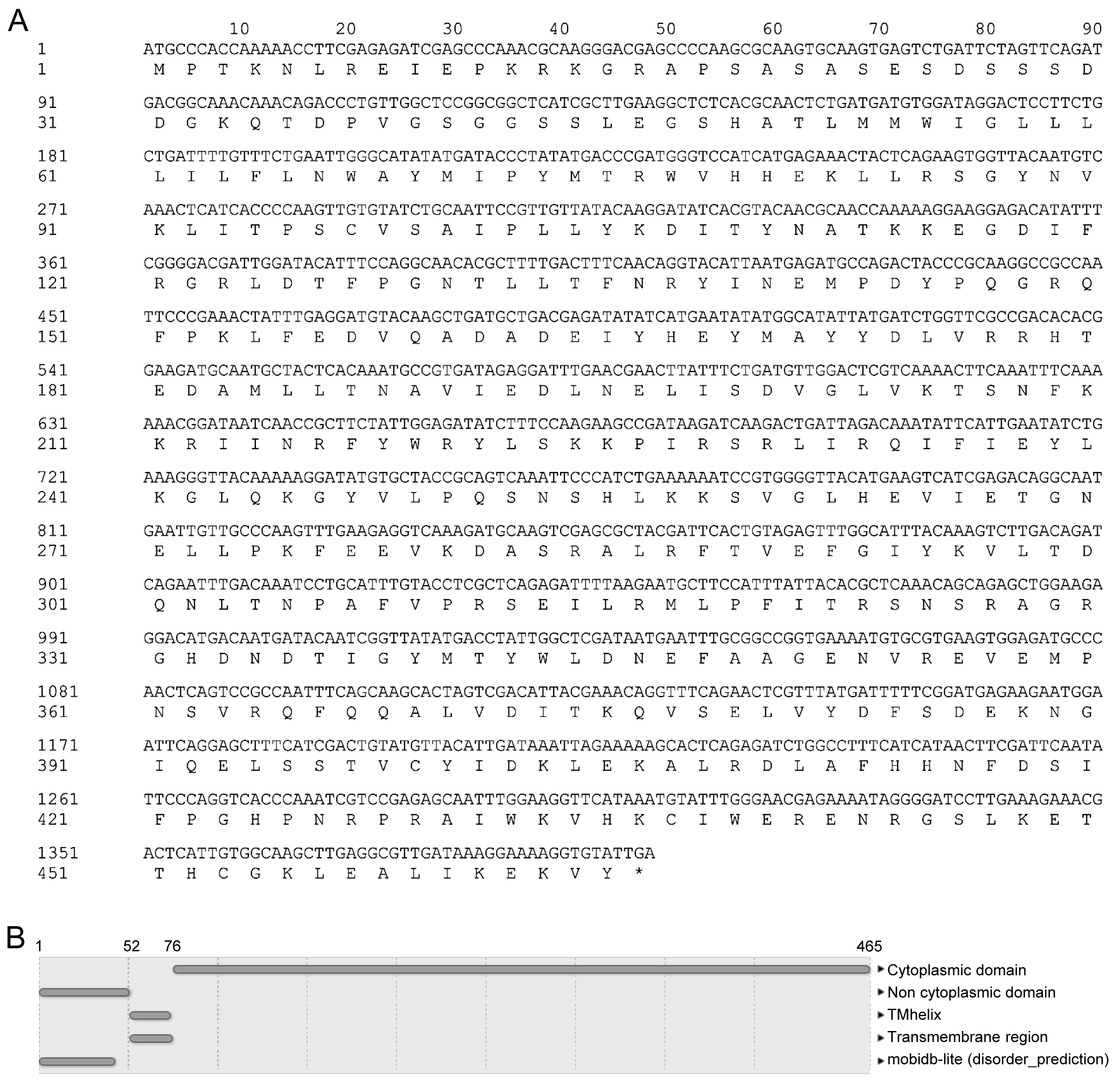

Supplement: Supplementary file 1 — Fig. S1 Sequence analysis of BcHBF1 in B. cinerea. (A) cDNA and deduced amino acid sequence of BcHBF1. (B) The deduced protein domains and functional sites of BcHbf1 based on InterProScan (http://www.ebi.ac.uk/interpro/scan.html) analyses. [file MPP-20-731-s001.tif]

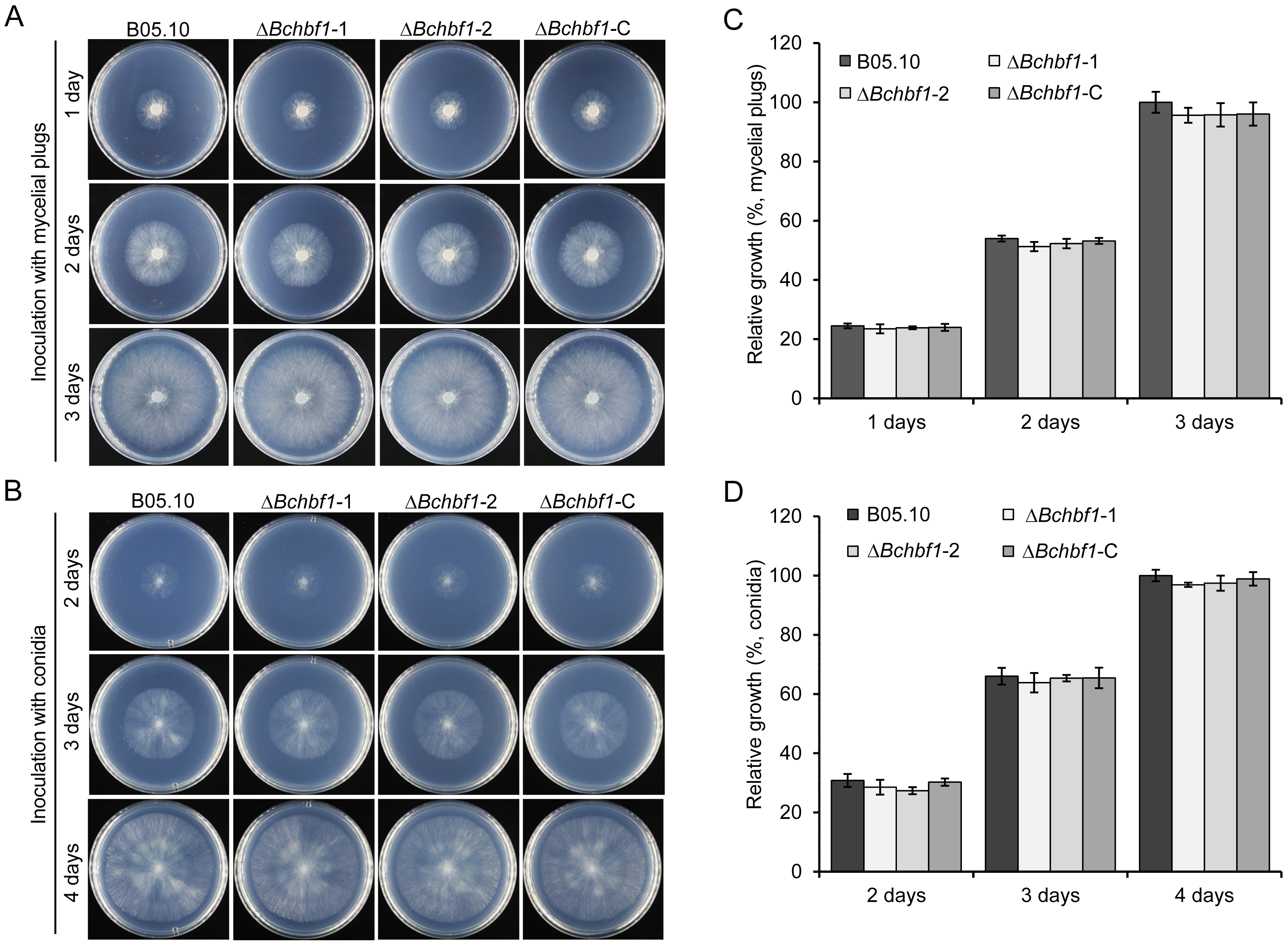

Supplement: Supplementary file 2 — Fig. S2 BcHBF1 is dispensable for B. cinerea mycelial growth. (A and B) BcHBF1 mediation of radial growth of mycelia (A) and conidial development (B) of the indicated B. cinerea strains on complete medium (CM) plates at 20 °C. (C and D) Quantification of radical growth of mycelia (C) and conidial development (D) of the indicated strains cultured on CM for 3 days and 4 days, respectively. Data represent means ± SD from three independent experiments with triplicate plates examined for each treatment. *, **, ***: significance at P < 0.05, P < 0.01 and P < 0.001, respectively. [file MPP-20-731-s002.tif]

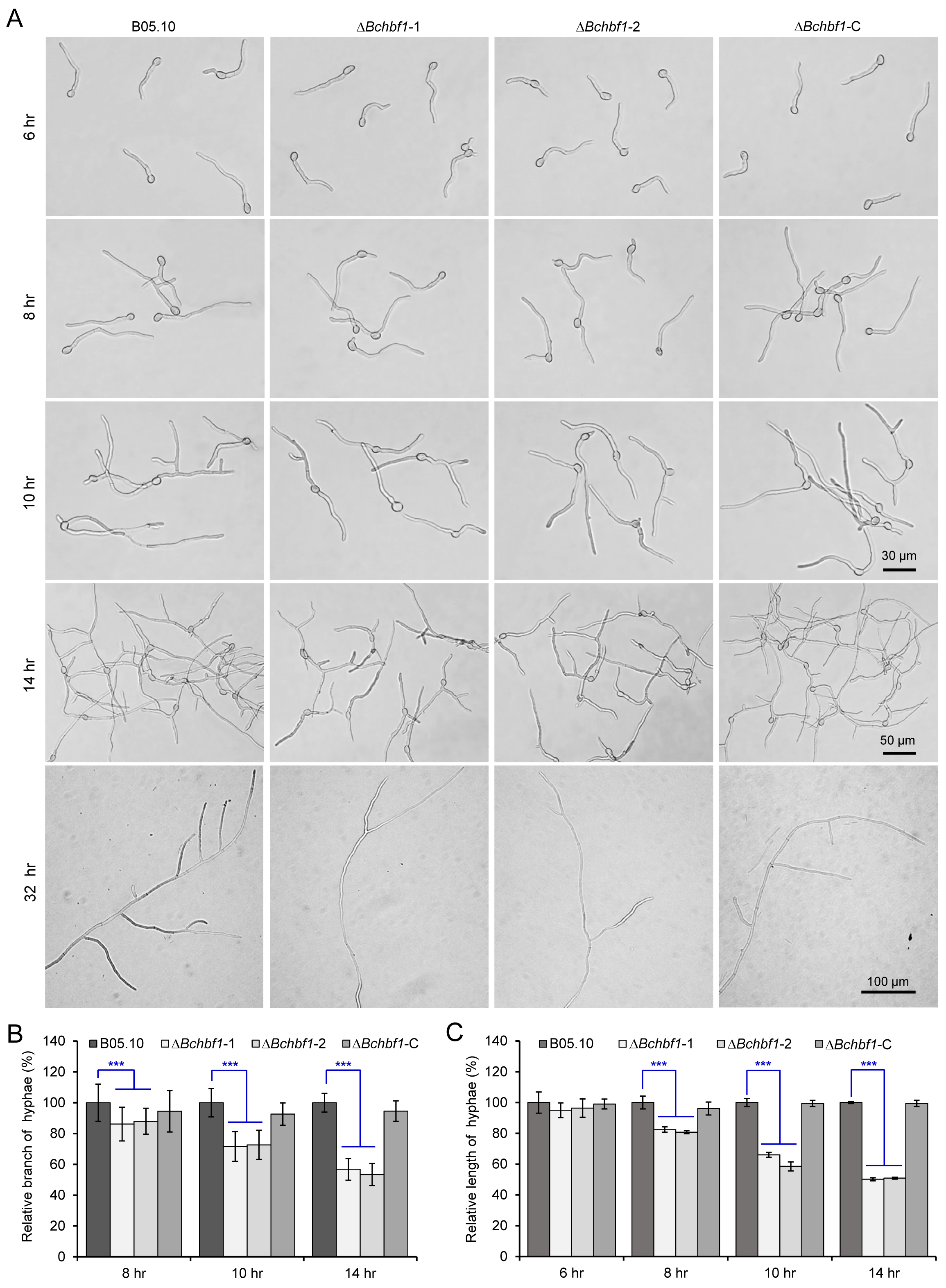

Supplement: Supplementary file 3 — Fig. S3 BcHBF1 is required for hyphal branching. (A) Droplets of conidial suspension (mixture of conidial suspension [1 × 10^5^ conidia mL for the time point 32 hpi, 1 × 10^6^ conidia mL for other time points] and PDB, vol: vol 1:1, 10 µl) of each strain were inoculated on glass slides and incubated at 20 °C. Germinated conidia were photographically documented at the indicated hpi. (B and C) Quantification of hyphal branching (B) and length (C) of the indicated strains at the indicated hpi. Data represent means ± SD from at least three independent experiments with triplicate slides examined for each treatment. *, **, ***: significance at P < 0.05, P < 0.01 and P < 0.001, respectively. [file MPP-20-731-s003.tif]
