# Supplementary material for: A novel Botrytis cinerea‐specific gene BcHBF1 enhances virulence of the grey mould fungus via promoting host penetration and invasive hyphal development
Source: Mol Plant Pathol. 2019 Apr 22;20(5):731–47. doi: 10.1111/mpp.12788 (PMC6637910; doi:10.1111/mpp.12788)
Supplement: Supplementary file 4 — Table S1 Primers used in this study. [file MPP-20-731-s004.docx]

**Table S1.** Primers used for this study.

| **Primer** | **Sequence（5’→3’）** | **Purpose** | |
| --- | --- | --- | --- |
| BcHBF1--UPF | ACTAGTCTGTGCCTCGTGGTGAAGAT | | Amplification of 5' flank for BcHBF1 |
| BcHBF1--UPR | GGTACCTGATGGCAATCCGTTGAAGT | | Amplification of 5' flank for BcHBF1 |
| BcHBF1--DNF | GTCGACATGGGTGGTTTATTCTATTTGC | | Amplification of 3' flank for BcHBF1 |
| BcHBF1--DNR | CTGCAGGAGTGGATGGCTCGTGAAG | | Amplification of 3' flank for BcHBF1 |
| BcHBF1--F | CGCAACTCTGATGATGTGGA | | Amplification of BcHBF1 |
| BcHBF1--R | GAGCGTGTAATAAATGGAAGCA | | Amplification of BcHBF1 |
| BcHBF1--CF | AAAGATCAAAGGATCGAATTCAGAAAGAGGCGATTGTGAAGT | | Amplification of *BcHBF1* complemented sequence |
| BcHBF1--CR | CCGGGTACCGAGCTCGAATTCATATGTTAGCGAACGAAGCAG | | Amplification of *BcHBF1* complemented sequence |
| BcHBF1--QF | GCTTCCATTTATTACACGCTCAA | | qRT-PCR for *BcHBF1* |
| BcHBF1--QR | TCTCCACTTCACGCACATTTT | | qRT-PCR for *BcHBF1* |
| BcActin--F | CATGGCTGGTCGTGATTTGA | | qRT-PCR for *BcAct* |
| BcActin--R | GAGGATTGACTGGCGGTTTG | | qRT-PCR for *BcAct* |
|  |  | |  |
